# Modulating the Swelling Behavior of Polymer Brushes via Interfacial Chemistry

**DOI:** 10.1002/anie.202507712

**Published:** 2026-05-19

**Authors:** Kuljeet Kaur, Sabrina Sant, Leon A. Smook, Sissi de Beer, Harm‐Anton Klok

**Affiliations:** ^1^ Institut des Matériaux and Institut des Sciences et Ingénierie Chimiques Laboratoire des Polyméres Bâtiment MXD Lausanne Switzerland; ^2^ Swiss National Center of Competence in Research (NCCR) Bio‐inspired Materials University of Fribourg Fribourg Switzerland; ^3^ Department of Chemistry Indian Institute of Technology, Gandhinagar Gandhinagar Gujarat India; ^4^ Department of Molecules & Materials MESA+ Institute University of Twente Enschede the Netherlands

**Keywords:** brush interface, polymer brush, surface chemistry, swelling

## Abstract

Polymer brushes composed of densely grafted end‐tethered polymer chains are often used in the solvent‐swollen state, for example, as boundary lubricants or non‐fouling surface coatings. For a given polymer brush in a specific solvent system, grafting density and polymer molecular weight are the principal structural parameters to tune swelling behavior. This report presents substrate surface chemistry as an additional parameter to control the swelling properties of polymer brushes generated via surface‐initiated atom transfer radical polymerization (SI‐ATRP). To uncover the impact of substrate surface chemistry, hydrophobic poly(tert‐butyl methacrylate) (PtBMA), and hydrophilic poly(2‐(dimethylamino)ethyl methacrylate) (PDMAEMA) brushes were grafted from substrates, which presented dipeptide and alkyl spacer tethered ATRP initiators and displayed water contact angles ranging from 48°–78°. Swelling of PtBMA brushes in a good solvent (THF) was found to be governed by polymer—solvent interactions, and substrate effects are negligible. In mixed solvents with a minority component that is a non‐solvent for the polymer, in contrast, substrate surface chemistry does influence swelling, and was found to correlate with the polarity of the dipeptide ATRP initiator presenting substrate. Results of the analysis of the swelling behavior of hydrophilic brushes in water also point toward an impact of substrate surface chemistry.

## Graphical abstract

Modulating substrate surface chemistry of surface‐grafted polymer brushes via variation of the chemical composition of spacers that anchor initiators for surface‐initiated atom transfer polymerizations allows to tune swelling of these thin polymer films.

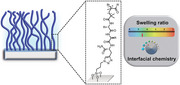

## 1 Introduction

The term polymer brush refers to a dense assembly of polymer chains that are tethered via one chain end to a solid surface [1, 2, 3, 4, 5, 6, 7, 8, 9, 10, 11]. When the grafting density of the brush, viz. the number of polymer chains per unit surface area, is sufficiently high, interchain interactions and excluded volume repulsions force the polymer chains to stretch in a direction normal to the surface into an extended chain conformation. Surface‐initiated polymerization techniques that use, for example, atom transfer radical polymerization (ATRP) [12, 13, 14], reversible addition‐fragmentation chain transfer polymerization (RAFT) [15, 16], nitroxide‐mediated polymerization (NMP) [17, 18], or other controlled/“living” polymerization processes provide access to polymer brushes with precisely controlled polymer molecular weights (film thicknesses), grafting densities, architectures, and functionalities [19, 20, 21].

Upon exposure to a good solvent, polymer brushes undergo swelling. The equilibrium thickness of a swollen polymer brush is the result of a balance between the solvent‐polymer interactions, and the entropic penalty associated with chain stretching. Swollen polymer brushes are often characterized in terms of their swelling ratio (*α*), which is defined as the ratio of the solvent‐swollen (*h*_swollen_) and dry (*h*_dry_) film thickness of the polymer brush. The high degree of solvation together with the stretched conformation of the polymer chains imparts swollen polymer brushes with unique properties such as an extraordinary resistance toward biofouling [22, 23, 24], and the ability to very effectively reduce friction between sliding surfaces [25, 26, 27, 28, 29], among others. The properties of polymer brushes in the solvent‐swollen state depend on the degree of swelling. As an example, experiments that investigated polystyrene brushes in toluene/isopropanol mixtures revealed a correlation between the swelling behavior of the brush and their lubrication properties [30]. In toluene‐rich mixtures, the polystyrene brushes were found to swell more, and lower friction coefficients were determined, as opposed to experiments that were conducted in isopropanol‐rich media in which the brushes swell less, and higher friction coefficients were measured.

As their properties in the solvent‐swollen state depend on the degree of swelling, it is essential to understand and be able to tune the swelling behavior of surface grafted polymer brushes. The swollen film thickness and swelling ratio of a polymer brush are typically attributed to 3 interdependent parameters, viz. the grafting density (*σ*) of the polymer brush, the molecular weight of the polymer grafts, and the solvent quality [3, 31, 32]. In addition to grafting density and polymer molecular weight, side chain dispersity has also been reported to impact the swelling behavior of polymer brushes [33]. For a polymer brush of a given chemical composition in a specific solvent, this leaves grafting density and polymer molecular weight as the principal structural parameters to tune swelling behavior. From a technological point of view, however, it may be desirable to be able to vary the swelling behavior of a polymer brush with a given chemical structure, grafting density, and molecular weight in one specific solvent. The number of approaches that allow to tune the swelling behavior of a polymer brush in a single solvent is very limited. One approach to control the swelling behavior of a polymer brush in a specific solvent medium involves the incorporation of (side chain) functional groups that will trigger conformational changes in the polymer grafts upon variation of environmental parameters such as pH, temperature, ion strength, or light [34]. While this represents an attractive avenue to tune the swelling properties of polymer brushes, it relies on changes in external, environmental parameters and thus does not allow to vary swelling behavior under a specific set of environmental conditions.

Another factor that may influence the conformation of chain end‐tethered polymers, and the thickness of polymer brushes in the swollen state, is the nature of the substrate onto which the polymer chains are tethered. Tuning the swelling behavior and properties of polymer brushes by varying the nature of the substrate, however, is impractical and limited in scope since different surfaces may require different chemistries to tether initiators or chain transfer agents, and since the number of surfaces that can be used to produce polymer brushes in a straightforward fashion is limited. To investigate the possible effects of surface chemistry on the swelling behavior of polymer brushes, this report explores a series of surface anchored initiators for ATRP, which differ in the chemical composition of the spacer that connects the ATRP initiating moiety and the underlying substrate. By incorporating dipeptide spacers composed of diserine, diglycine or diphenylalanine, or alkyl spacers consisting of 3–9 methylene units, ATRP initiator‐modified substrates covering a range of surface polarities were obtained. These substrates have been used to graft hydrophobic poly(tert‐butyl methacrylate) (PtBMA) brushes (*h*_dry_ > 100 nm) and hydrophilic poly(2‐(dimethylamino)ethyl methacrylate) (PDMAEMA) brushes (*h*_dry_ > 60 nm), which were subsequently employed to uncover possible effects of substrate surface chemistry on the swelling properties of polymer brushes in both organic solvents as well as in aqueous media.

## 2 Results and Discussion

**FIGURE 1:**
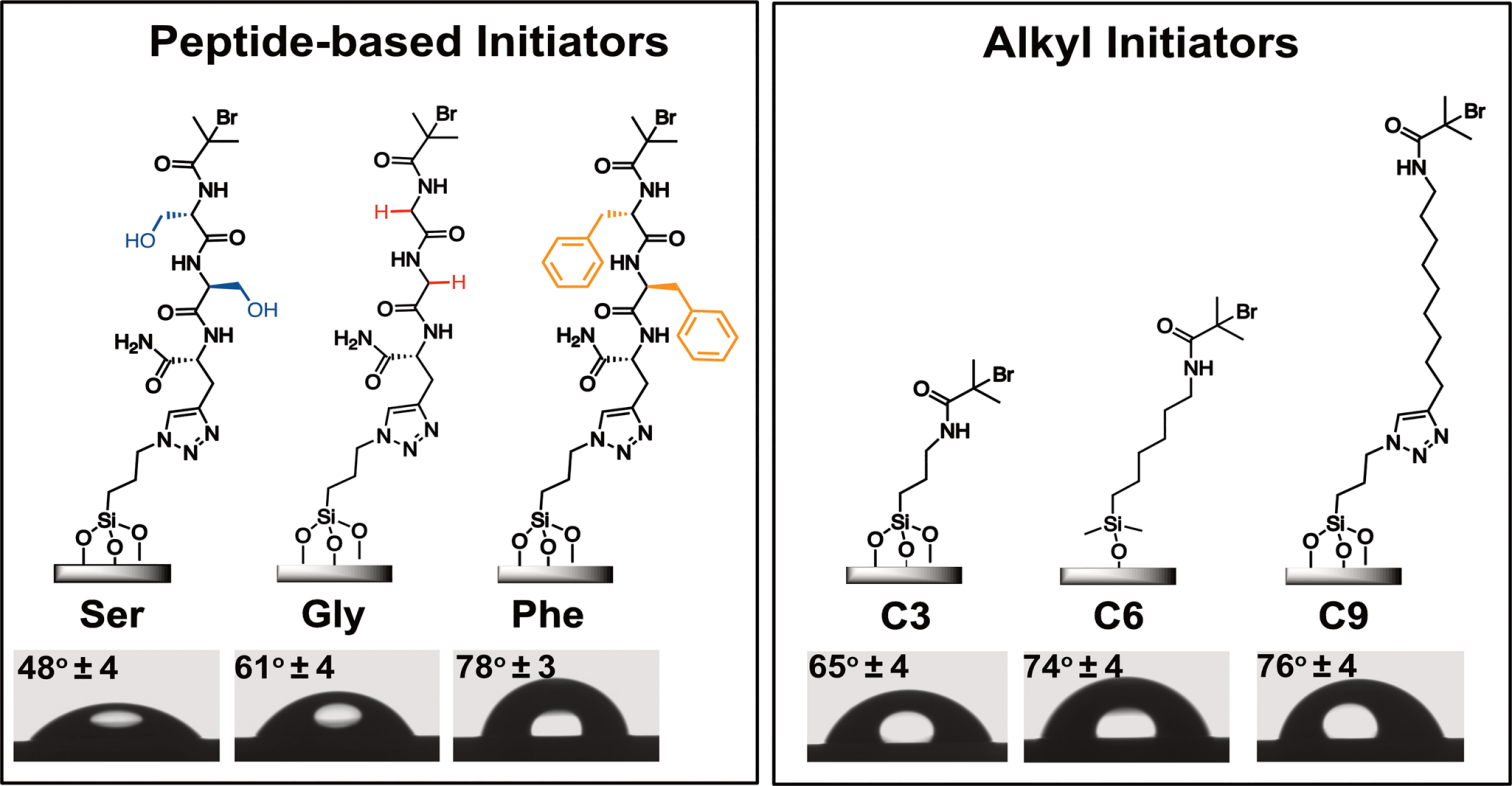
Chemical structures of the dipeptide‐based (**Ser**, **Gly**, **Phe**), and alkyl spacer tethered ATRP initiators (**C3**, **C6**, **C9**) used in this study, and the water contact angles of the different ATRP initiator‐modified surfaces.

The experiments reported in this paper were conducted on polymer brush films grafted from silicon substrates via surface‐initiated atom transfer radical polymerization (SI‐ATRP). To probe the influence of surface chemistry on the swelling behavior of surface‐anchored polymer brushes, two series of ATRP initiator‐modified surfaces were designed and prepared (Figure 1).

The first group of initiators contains a dipeptide spacer that connects the ATRP initiating 2‐bromoisobutyramide group with the alkoxysilane surface anchor. The incorporation of a dipeptide spacer is attractive as it allows the creation of a diverse variety of structurally analogous initiators by variation of the constituent amino acids. Since amino acids are available with side chain functional groups that vary from polar to non‐polar, this provides access to silicon substrates that present a broad range of surface chemistries and polarities. The three peptide‐based initiators in Figure 1 contain serine (**Ser**), glycine (**Gly**), or phenylalanine (**Phe**), which were selected as representative amino acids to prepare dipeptides that range from hydrophilic (**Ser**) to hydrophobic (**Phe**). The second family of ATRP initiators that was investigated incorporates an alkyl spacer that connects the 2‐bromoisobutyramide group and the organosilane surface anchoring group (Figure 1). Three different initiators containing three (**C3**), six (**C6**), or nine (**C9**) methylene units were designed and prepared in order to systematically vary the hydrophobicity of the ATRP initiator‐modified silicon surface [35].

Peptide‐based initiator‐functionalized surfaces were prepared by copper catalyzed azide–alkyne cycloaddition (CuAAC) of the corresponding alkyne‐modified dipeptides onto azide‐modified silicon wafers (Scheme S1). These dipeptides were synthesized using standard Fmoc solid phase peptide synthesis (details are provided in the Supporting Information). All dipeptides incorporate a 2‐bromoisobutyl group, which serves as initiator for ATRP, at the N‐terminus. As the C‐terminal residue, a propargylglycine unit was installed to allow immobilization of the peptide onto the silicon surfaces. The resulting ATRP initiator functionalized surfaces were characterized by x‐ray photoelectron spectroscopy (XPS) and water contact angle analysis. XPS survey scans and C1s, N1s, and Br3rd high resolution spectra of the peptide‐initiator modified silicon surfaces are presented in Figure S1. The C1s high resolution scans can be fitted with 3 residuals that can be assigned to C─O/C─N, C═O, and C─C carbon atoms. The N1s high resolution scans of the peptide‐modified surfaces can be resolved into two signals, corresponding to the N─N═N nitrogen atom from the triazole group at ∼ 401.5 eV, and the R_2_NH/R‐NH_2_ peptide nitrogen atoms and the C─N═N nitrogen atoms of the triazole group at ∼ 400 eV. Water contact angle measurements highlighted the pronounced dependence of the wettability of the peptide‐based initiator‐modified surfaces toward subtle variations in the amino acid composition of the spacer. While for a silicon surface modified with the serine‐based initiator a water contact angle of 48° ± 4° was determined, silicon substrates that present the glycine or phenylalanine‐spacer based initiators revealed water contact angles of 61° ± 4°, respectively 78° ± 3° (Figure 1).

Silicon substrates presenting ATRP initiators tethered via alkyl spacers of different lengths (**C3**, **C6,** and **C9**) were prepared via literature procedures (see Supporting Information) [36, 37, 38, 39]. Water contact angle analysis of the **C3**, **C6**, and **C9** ATRP initiator modified surfaces indicated water contact angles of 65° ± 4°, 74° ± 4°, and 76° ± 4° (Figure 1). Comparison of the water contact angles measured on the **Ser**, **Gly**, and **Phe** modified silicon substrates with those of the **C3**, **C6**, and **C9** surfaces shows that tuning the amino acid composition of the peptide spacer represents a more powerful strategy to modulate the wettability of water droplets on the silicon substrates as compared to varying the number of methylene units in the alkyl spacer based ATRP initiators. Whereas variation of the alkyl spacer length from 3 to 9 methylene groups allows to change the water contact angle from 65° ± 4° to 76° ± 4°, tuning the amino acid composition of the peptide spacer provides access to a much wider range of water contact angles, viz. 48° ± 4°–78° ± 3°.

**SCHEME 1:**
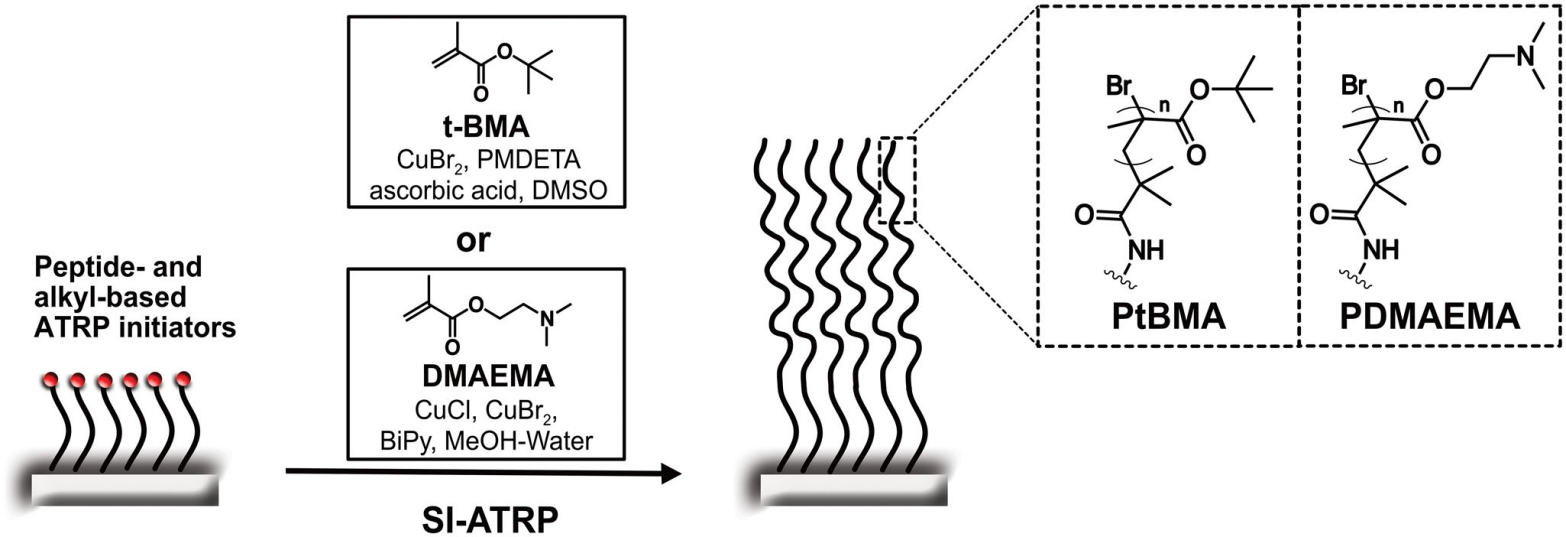
Synthesis of PtBMA and PDMAEMA brushes via SI‐ATRP.

Silicon substrates presenting the peptide and alkyl spacer‐based ATRP initiators were subsequently used for the synthesis of hydrophobic poly(tert‐butyl methacrylate) (PtBMA), and hydrophilic poly(2‐(dimethylamino)ethyl methacrylate) (PDMAEMA) brushes via SI‐ATRP (Scheme 1). PtBMA brushes were prepared in DMSO at room temperature via activators regenerated by electron transfer (ARGET) ATRP using CuBr_2_ / PMDETA/ascorbic acid as the catalyst system. This resulted in peptide‐anchored brushes with dry film thicknesses of 104 ± 9 nm (**Ser‐PtBMA**), 122 ± 13 nm (**Gly‐PtBMA**), and 161 ± 37 nm (**Phe‐PtBMA**), respectively. Polymerization of tBMA from the alkyl‐spacer‐based ATRP initiators using the same procedure generated films with dry thicknesses of 167 ± 28 nm (**C3‐PtBMA**), 111 ± 4 nm (**C6‐PtBMA**), and 155 ± 19 nm (**C9‐PtBMA)**. DMAEMA was polymerized from peptide‐functionalized wafers using a conventional SI‐ATRP protocol [40] with CuCl/CuBr_2_/2,2′‐bipyridine as the catalyst to provide PDMAEMA brushes with dry film thicknesses of 109 ± 3 nm (**Ser‐PDMAEMA**), 130 ± 45 nm (**Gly‐PDMAEMA**), and 66 ± 28 nm (**Phe‐PDMAEMA**). Using the **C3**‐, **C6**‐, and **C9**‐based ATRP initiator‐functionalized silicon substrates, PDMAEMA brushes with dry film thicknesses of 143 ± 39 nm (**C3‐PDMAEMA**), 147 ± 1 nm (**C6‐PDMAEMA**), and 134 ± 35 nm (**C9‐PDMAEMA**) were obtained with this procedure. Table S1 provides the structural characteristics, including the grafting density (*σ*), the reduced grafting density (*Σ*), and the polymer graft molecular weight (*M*_n_) of the polymer brushes. For all samples listed in Table S1 *Σ* > 60, indicating that the brushes are well in the brush regime (the “*true brush*” regime is typically defined as *Σ* > 5) [11].

**FIGURE 2:**
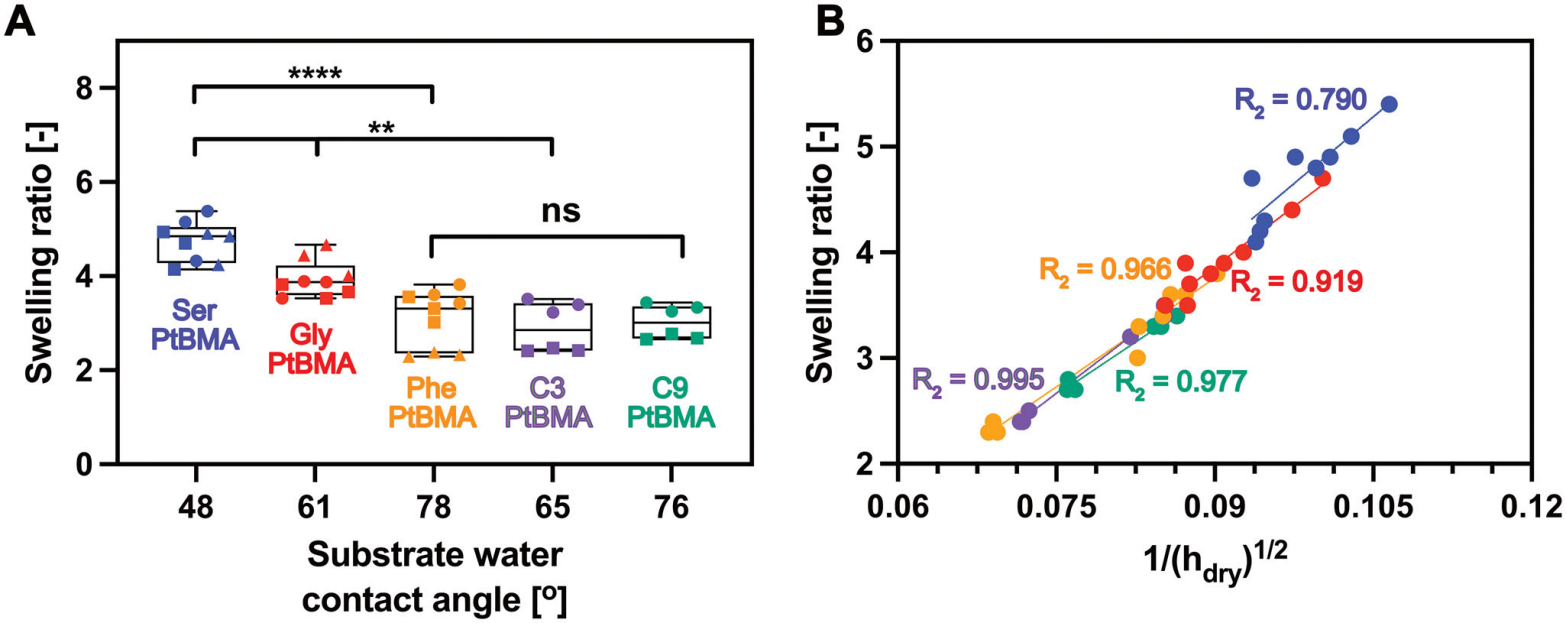
(A) Box plot presentation of the swelling ratios for PtBMA brushes grown from peptide‐based and alkyl‐based initiators in THF as a function of the water contact angle of the corresponding silicon substrates. Box plots represent the interquartile range of data between 25th and 75th percentile, and whisker represents spread of data between maximum and minimum including outliers. Median is represented as horizontal lines. ** Indicates *p* < 0.05, **** indicates *p* < 0.0001, and ns indicates “not significant” for comparisons among the groups indicated, as determined by a one‐way analysis of variance (ANOVA) with a Tukey comparison post hoc test. (B) Swelling ratios plotted as a function of 1/√*h*_dry_. Each data point (solid dots) in plots **A** and **B** corresponds to one value of swelling ratio per brush sample. The data points belonging to the same batch are represented using same symbols in plot **A** for clarity.

The film thickness of solvent‐swollen polymer brushes can be determined using a number of techniques, including ellipsometry, x‐ray and neutron reflectometry as well as atomic force microscopy (AFM) [4, 41]. The use of AFM imaging and force distance measurements, however, has been shown to under‐, respectively, overestimate swollen film thicknesses [42]. For the experiments in this paper, ellipsometry was used to measure both the dry (*h*_dry_) and solvent‐swollen thickness (*h*_swollen_) of the polymer brushes. The ratio of the swollen film thickness and the dry film thickness provides the swelling ratio (*α* = *h*_swollen_/*h*_dry_), which was used to compare the different polymer brush samples. The experiments were performed using nine independent samples (from three separate synthetic batches), respectively, six independent samples (from two separate synthetic batches) for the peptide and alkyl‐spacer anchored polymer brushes. In a first series of experiments, the swollen film thicknesses of PtBMA brushes were analyzed in THF, which is a good solvent for the polymer grafts. Figure 2A presents the swelling ratios that were determined in THF for PtBMA brushes grown from the peptide and alkyl initiator‐modified surfaces. The corresponding dry and swollen film thicknesses are summarized in Figures S2 and S3. For a PtBMA brush grafted from a **Ser**‐based initiator surface, a swelling ratio of 4.7 ± 0.4 was measured. Changing the amino acid composition of the dipeptide spacer resulted in significant changes in the swelling ratio. While for the **Gly**‐tethered PtBMA brush a swelling ratio of 3.9 ± 0.4 was obtained in THF, analysis of the **Phe**‐anchored PtBMA brush revealed a swelling ratio of 3.1 ± 0.6 in the same solvent. Analysis of **C3** and **C9** tethered PtBMA brushes in THF afforded swelling ratios of 2.9 ± 0.5 and 3.0 ± 0.4.

Figure 2A indicates significant differences in swelling ratio across **Ser**, **Gly**, and **Phe** grafted PtBMA brushes in THF. While the preparation of the initiator‐modified substrates was conducted with great care, small variations in ATRP initiator surface concentration cannot be completely ruled out. In addition, there may be differences in initiator efficiency across the various peptide and alkyl‐anchored ATRP initiators toward the polymerization of tBMA. Both of these factors can result in variations of grafting density between the different peptide and alkyl‐spacer tethered PtBMA brushes. Since the swelling ratio (*α*) of a densely grafted brush depends on the grafting density (*σ*) as *α* ∼ 1/√*σ* [43], it is important to deconvolute effects that are due to differences in grafting density from possible substrate surface chemistry effects. To disentangle these two possible contributions, Figure 2B plots the swelling ratios that were determined for the peptide and alkyl‐spacer attached PtBMA brush samples as a function of 1/√*h*_dry_ (since *h*_dry_ is directly proportional to *σ* [4]). Figure 2B shows that the swelling ratios of most of the investigated PtBMA brush samples, with the exception perhaps of PtBMA brushes grafted from the most hydrophilic **Ser** initiator‐modified surface, linearly scale with 1/√*h*_dry_. This indicates that the differences in swelling ratio that are observed in the good solvent THF can be attributed to small sample‐to‐sample variations in grafting density.

**FIGURE 3:**
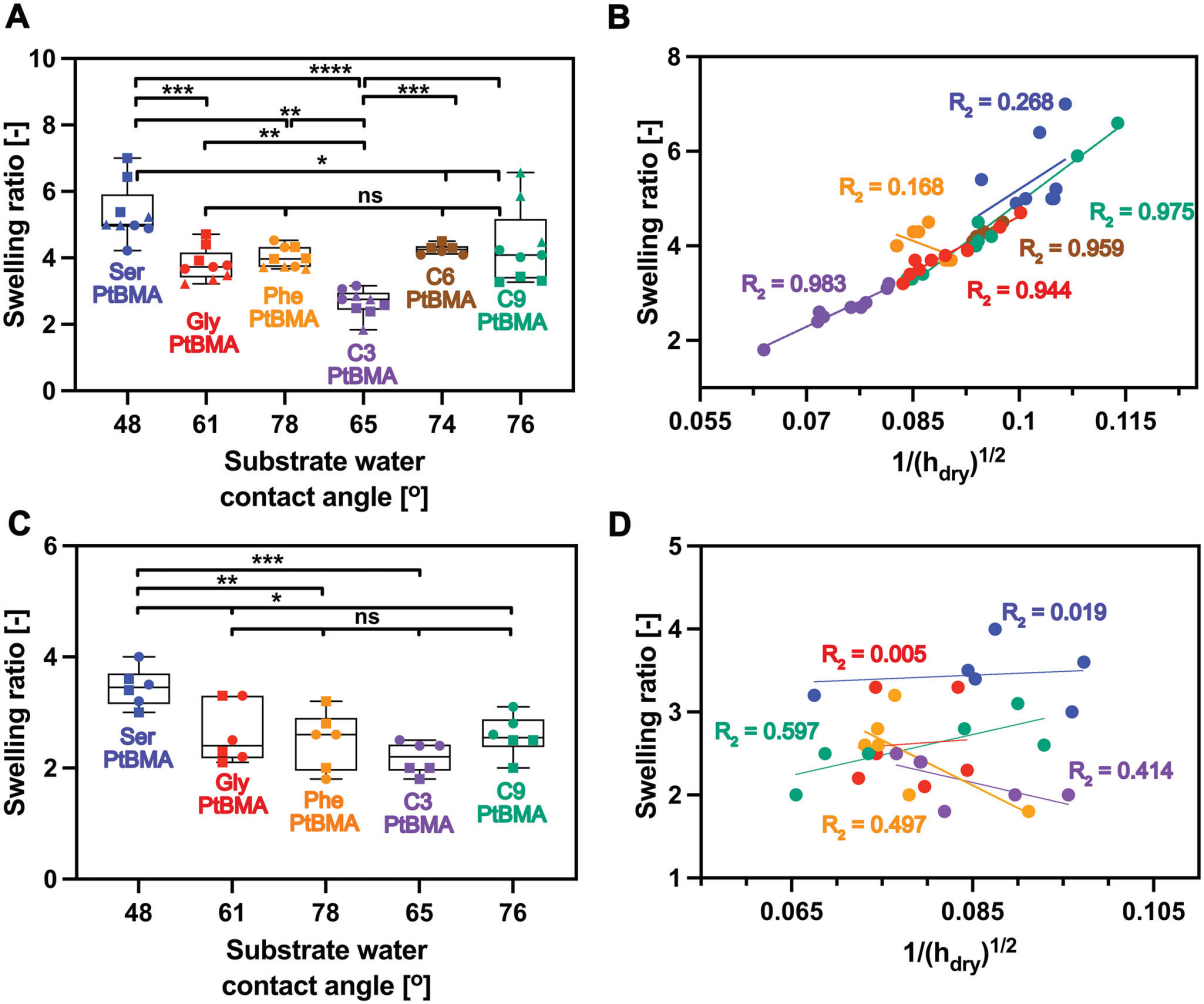
Box plot presentation of the swelling ratios for PtBMA brushes grown from peptide‐based and alkyl‐based initiators in (A) THF/water (95/5, *v*/*v*) and (C) acetone/water (95/5, *v*/*v*), as a function of the water contact angle of the corresponding silicon substrates. Box plots represent the interquartile range of data between 25th and 75th percentile and whisker, represents spread of data between maximum and minimum including outliers. Median is represented as horizontal lines. * Indicates *p* < 0.05, ** indicates *p* < 0.005, *** indicates *p* < 0.0005, **** indicates *p* < 0.0001, and ns indicates “not significant” for comparisons among the groups indicated, as determined by a one‐way analysis of variance (ANOVA) with a Tukey comparison post hoc test. Swelling ratios plotted as a function of 1/√*h*_dry_ for the experiments conducted in (B) THF/water (95/5, *v*/*v*) and (D) acetone/water (95/5, *v*/*v*). Each data point (solid dots) in plots A, B, C, and D, corresponds to one value of swelling ratio per brush sample. The data points belonging to the same batch are represented using same symbols in plot A and C for clarity.

Next, the swelling behavior of the peptide and alkyl‐spacer grafted PtBMA brushes was studied in poorer solvent systems using THF/water (95/5, *v*/*v*) and acetone/water (95/5, *v*/*v*). Figure 3 presents the swelling ratios determined for the investigated peptide and alkyl‐anchored PtBMA brushes in these two solvent systems, and also plots *α* versus 1/√*h*_dry_ for the studied samples.

The results presented in Figure 3A indicate significant differences in swelling ratio between **Ser**, **Gly,** and **Phe‐PtBMA**, as well as between **Ser‐PtBMA** and the alkyl‐tethered PtBMA brushes, and **Gly‐PtBMA** and **C3‐PtBMA** in THF/water 95/5 (*v*/*v*). Figure 3B shows that the swelling behavior of **Gly**, **C3, C6**, and **C9‐PtBMA** samples is captured well by the 1/√*h*_dry_ scaling dependence, indicating that differences in swelling ratio between these samples are mainly governed by variations in grafting density. The swelling ratios of the **Ser** and **Phe‐PtBMA** samples in contrast do not follow this scaling law dependence, indicating that the swelling behavior of these samples in this poorer solvent system (as compared to pure THF) is not solely dictated by variations in grafting density. The swelling ratio of the PtBMA brush grafted from the more hydrophilic **Ser** initiator in THF/water 95/5 (*v*/*v*) is significantly higher as compared to that for the same brush grafted from the more hydrophobic **Phe**‐initiator substrate, indicative of a contribution of the chemical composition of the ATRP initiator‐modified substrate to the swelling behavior. The impact of substrate surface chemistry on the swelling behavior of the PtBMA brushes becomes even more pronounced when the solvent quality is further decreased, and swelling ratios are determined acetone/water 95/5 (*v*/*v*) (Figure 3C,D). Analysis of the swelling ratios in this solvent system reveals significant differences in swelling ratio across the **Ser**, **Gly**, and **Phe‐PtBMA** brushes, as well as for **Ser** and **Gly‐PtBMA** as compared to **C3** and **C9‐PtBMA**. The data analysis presented in Figure 3D, however, shows that for none of these brushes the swelling behavior of the samples is described well by the scaling law dependence that would account for variations in grafting density across the samples. This suggests that in acetone/water (95/5, *v*/*v*) the observed differences in grafting density (which for **Ser**, **Gly**, and **Phe‐PtBMA** correlate with the water contact angle of the initiator‐modified substrate) are dominated by substrate surface chemistry. Taken together, the results presented in Figures 2 and 3 indicate that swelling of PtBMA brushes in a good solvent for the polymer grafts (THF) is essentially exclusively governed by polymer—solvent interactions, and substrate effects are negligible. In mixed solvent systems that include a minority component that is a non‐solvent for the polymer grafts, in contrast, substrate surface chemistry does influence swelling behavior as illustrated in Figure 3B (for **Ser‐PtBMA** and **Phe‐PtBMA**) and in Figure 3D (for all investigated samples). Under these conditions, the minority solvent component might preferentially segregate at, and wet the polymer brush—substrate interface, as described theoretically by Johner and Marques [44], and experimentally by Gallagher et al. [45]. If the solvent that segregates at the polymer brush—substrate interface (water in this case) is immiscible with the polymer (PtBMA in this case) then repulsive interactions are expected to result in chain stretching and increased swelling of the brushes.

**FIGURE 4:**
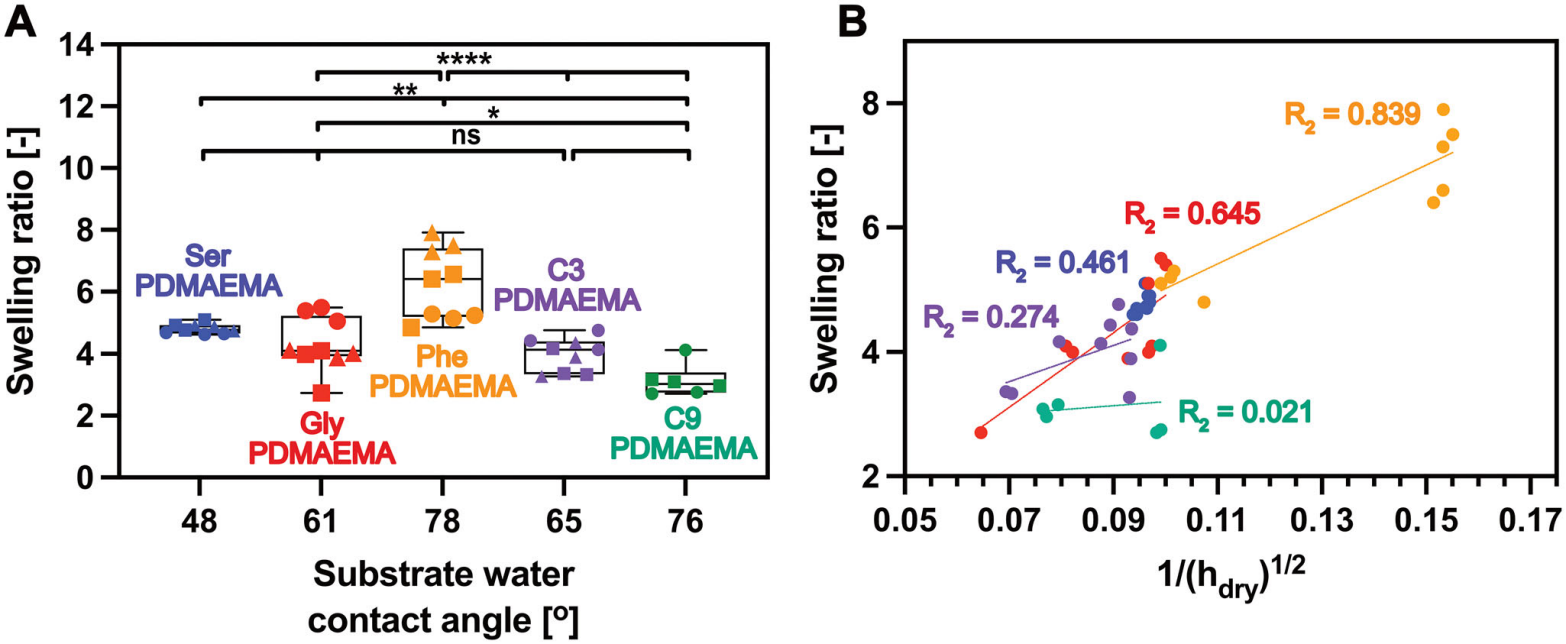
Swelling ratios of PDMAEMA brushes grafted from peptide and alkyl‐based initiators in 10 mM PBS buffer. Each data point represents one value of swelling ratio per wafer. Box plots represent the interquartile range of data between 25th and 75th percentile and whisker, representing spread of data between maximum and minimum including outliers. Median is represented as horizontal lines. * Indicates *p* < 0.05, ** indicates *p* < 0.005, **** indicates *p* < 0.0001, and ns indicates “not significant” for comparisons among the groups indicated, as determined by a one‐way analysis of variance (ANOVA) with a Tukey comparison posthoc test. (B) Swelling ratios of the investigated samples plotted as a function of their respective 1/√*h*_dry_. The data points belonging to the same batch are represented using same symbols in plot **A** for clarity.

In a final set of experiments, the swelling behavior of PDMAEMA brushes grown from the different peptide and alkyl‐initiator‐modified substrates was studied in 10 mM PBS buffer using the same methodology as applied to the PtBMA brushes. Figure 4 presents the measured swelling ratios of the peptide and alkyl‐grafted PDMAEMA brushes, and also plots the swelling ratios of the investigated samples as a function of 1/√*h*_dry_ (the corresponding dry and swollen film thicknesses are included in Figures S2 and S3). As indicated by the results in Figure 4B, the swelling ratios of the PDMAEMA brushes in water do not follow the 1/√*h*_dry_ scaling law dependence. This is expected as swelling of charged polyelectrolyte brushes is independent of grafting density, and dominated by osmotic pressure [46]. It also indicates that differences in swelling ratio (such as e.g., between **Ser** and **Phe‐PDMAEMA**) are due to other parameters, including putative effects of substrate surface chemistry. The results of the swelling behavior analysis in water, however, are less intuitive as compared to those in THF (or THF/water, or acetone/water), and will require further work to fully delineate the specific contributions of substrate surface chemistry in those media.

## 3 Conclusion

This report has presented substrate surface chemistry as an additional parameter that can impact the swelling properties of polymer brushes generated via SI‐ATRP. Anchoring initiators via peptide or alkyl spacers of different chemical compositions allows to prepare substrates for SI‐ATRP with water contact angles ranging from 48°–78°. To uncover the impact of substrate surface chemistry, the swelling behavior of hydrophobic poly(tert‐butyl methacrylate) (PtBMA) and hydrophilic poly(2‐(dimethylamino)ethyl methacrylate) (PDMAEMA) brushes grafted from substrates modified via ATRP initiators tethered via diserine, diglycine, or diphenylalanine spacers, or via linkers that incorporate 3, 6, or 9 methylene units, was investigated. Swelling of PtBMA brushes in a good solvent (THF) was found to be essentially exclusively governed by polymer—solvent interactions, and substrate effects are negligible. In mixed solvents with a minority component that is a non‐solvent for the polymer, in contrast, substrate chemistry does influence swelling, and was found to correlate with the polarity of the dipeptide ATRP initiator presenting substrate. Results of the analysis of the swelling behavior of hydrophilic brushes in water also point toward an impact of substrate surface chemistry, however, are less intuitive as compared to those in (mixed) organic media, and will require further work to fully delineate the specific contributions of substrate surface chemistry. The strategy presented here provides an avenue to modulate swelling of a given polymer brush in a single, specific solvent system, as well as a new approach to tune the solvent‐responsiveness of these thin films. This complements existing approaches that typically rely on variation in structural parameters (grafting density and polymer molecular weight) or solvent (composition) to tune swelling behavior. As polymer brushes are often used in the solvent‐swollen state, and their properties depend on the swelling ratio, additional methodologies to tune the swelling behavior of these thin polymer films are not only of fundamental interest, but also technologically relevant.

## Conflicts of Interest

The authors declare no conflicts of interest.

## Supporting information




**Supporting File**: Materials, methods, experimental procedures, XPS spectra, mass spectra, ^1^H, and ^13^C NMR spectra.

## Data Availability

The data that support the findings of this study are openly available in the Zenodo repository at https://doi.org/10.5281/zenodo.20121151.
