# Supplementary material for: Modulating the Swelling Behavior of Polymer Brushes via Interfacial Chemistry
Source: Angew Chem Int Ed Engl. 2026 May 19;65(29):e07712. doi: 10.1002/anie.202507712 (PMC13360554; doi:10.1002/anie.202507712)
Supplement: Supplementary file 1 — Supporting File: Materials, methods, experimental procedures, XPS spectra, mass spectra, 1H, and 13C NMR spectra. [file ANIE-65-e07712-s001.pdf]

Supporting Information

Modulating the Swelling Behavior of Polymer Brushes via Interfacial Chemistry

*Kuljeet Kaur,[1,2,3] Sabrina Sant,[1,2] Leon A. Smook,[4] Sissi de Beer,[4] and Harm-Anton Klok[1,2]**

1. Institut des Matériaux and Institut des Sciences et Ingénierie Chimiques,

Laboratoire des Polymères, École Polytechnique Fédérale de Lausanne (EPFL)

Station 12, CH-1015 Lausanne, Switzerland

2. Swiss National Center for Competence in Research (NCCR) Bio-inspired Materials

University of Fribourg, Chemin des Verdiers 4, CH-1700 Fribourg, Switzerland

3. Department of Chemistry, Indian Institute of Technology

Gandhinagar, Palaj, Gandhinagar, Gujarat-382355, India

4. Department of Molecules & Materials, MESA+ Institute, University of Twente, Enschede 7500AE,

The Netherlands

kuljeet.kaur@epfl.ch, sabrina.sant@epfl.ch, l.a.smook@utwente.nl, s.j.a.debeer@utwente.nl, harm-anton.klok@epfl.ch

CORRESPONDING AUTHOR: Email: harm-anton.klok@epfl.ch; Phone: + 41 21 693 4866

MATERIALS

All reagents and solvents were used as received unless noted otherwise. Rink amide AM resin (loading 0.7 g/mmol), Fmoc protected amino acids, Fmoc-Glycine (Gly), Fmoc-L-Serine (Ser), Fmoc-L-Phenylalanine (Phe), and (2-(1*H*-benzotriazol-1-yl)-1,1,3,3-tetramethyluronium hexafluorophosphate (HBTU) were purchased from Iris Biotech. Fmoc-L-propraglycine (Pra) (97%) was purchased from ABCR. 2-Bromoisobutyric acid (BiB), 2-bromoisobutryl bromide, *N,N*-diisopropylethylamine (DIEA), trifluoroacetic acid (TFA), (3-amino propyl)triethoxy silane (APTES) and triisopropylsilane (TIPS) were purchased from Sigma Aldrich. 3-(Chloropropyl)triethoxy silane was purchased from Chemie Brunschwig. Sodium azide, tetrabutylammonium bromide (TBAB) and N,N,N′,N″,N″-pentamethyldiethylenetriamine (PMDETA) were purchased from Sigma Aldrich. Tris((1-benzyl-triazolyl) methyl) amine (TBTA) (99%) was purchased from Adipogen SA. 10-Undec-1-ol was purchased from Fisher Scientific. Solid phase extraction (SPE) columns (Sep-Pak C18 6 cc Vac Cartridges, 1g sorbent, 55-105 μm) used for peptide purification were purchased from Waters corporation. Anhydrous DMSO (99.9%) for click reactions was purchased from ABCR, and used without any purification. Copper(I)bromide (99.995+%), copper(II)bromide (99.999%), tert-butylmethacrylate (tBMA), and 2-(dimethylamino)ethyl methacrylate (DMAEMA) were purchased from Sigma Aldrich, and were passed through basic alumina before polymerization. MilliQ and DI water were obtained from Millipore Direct-Q 5 ultrapure, and E-POD Pure Water Remote Dispensor systems (Merck), respectively. THF, toluene, and $CHCl_3$ were obtained from a PureSolv MD5 purification system by Inert corporation. Acetonitrile was dried over molecular sieves (3 Å) for 48 h before use. Silicon wafers (polished on one side, 100/P/DS/1-10) were obtained from, and diced into 8.0 x 5.5 mm sized rectangles, in the Centre of MicroNanotechnology at EPFL. All silicon wafers used are covered by a ~ 2 nm silicon oxide layer based on ellipsometry analysis.

METHODS

Plasma treatment of silicon wafers: Silicon wafers were cleaned using a Femto O_2 Plasma System (200 W, Diener Electronic) with power and gas flow set to 190 W and 10 (l_n/min), respectively.

Nuclear Magnetic Resonace (NMR) Spectroscopy: ^1H and ^{13}C NMR spectra were recorded on a Bruker AVANCE-400 Ultra shield spectrometer. Deutrated solvents (choloroform, $CDCl_3$) were used and chemical shifts (δ) are reported in ppm relative to internal solvent resonances. NMR data was analysed using MNOVA software from Mestrelab.

XPS: XPS analysis was carried out using an Axis Ultra instrument from Kratos Analytical equipped with a conventional hemispheric analyzer. The X-ray source employed was a monochromatic Al Kα (1486.6 eV) source operated at 100 W and 10^{-9} mbar. MultiPak software was used to analyze the atomic concentrations with the relative sensitivity factors (RSF) provided in the same software. Deconvolution of high-resolution XPS spectra to analyze chemical states was performed using CasaXPS software. Calibration of XPS was done by setting the C1s signal at 284.8 eV

Ellipsometry: The thicknesses of dry and swollen brushes were measured using a SemiLab SEA2000 ellipsometer equipped with a liquid cell. The incident angle and the wavelength for the light microspot were 70° and 634.72 nm, respectively. Ellipsometric angles Ψ and Δ were recorded between 300 and 700 nm. Data fitting was performed using SEA software (v1.6.1) (Semilab, Hungary) using Levenerg-Marquardt algorithim (LMA). The refractive index (n) of polymer film is determined by Cauchy equation ($n(\lambda) = A + B/\lambda^2$) where λ is the wavelength of the incident light, and A and B are fitting parameters that refer to refractive index of material at long and short wavelengths, respectively.

Dry film thicknesses (h_{dry}) were modeled using a four-layer box optical model with silicon / silicon oxide / polymer brush / air layers. Refractive indices used for Si (n = 3.881) and SiO_2 (n = 1.457) layer were provided by SEA v1.6.1 software. Polymer film thicknesses were estimated by keeping Cauchy's A parameter constant at 1.464[1] and 1.513[2] for PtBMA and PDMAEMA brushes, and by the varying film thickness and parameter B to match the recorded experimental data (Ψ and Δ). A good fit is determined by $R^2 > 0.99$ and mean square error (MSE) < 0.5. A minimum of 4 data points per wafer were analyzed and the thickness values were obtained as an average.

Swollen film thicknesses ($h_{swollen}$) were determined on polymer brush samples that were immersed in the solvent inside a liquid cell, by analyzing one spot per wafer. Instrument parameters like incident angle and wavelength were same as for measuring dry film thickness. Swollen film thickness was modeled using a four-layer optical model with silicon / silicon oxide / polymer brush / solvent layers. Refractive indices used for Si, SiO_2, and the pure solvent layers were provided by SEA v1.6.1 software (**Supporting Information Table S2**). Fitting of the swollen film thickness was performed using 'gradient model' provided by SEA v1.61 software, and polymer brush film was assumed to have a parabolic density profile (or $c(z) = a(z-z_0)^2 + c_0$ where c is top layer concentration and z is phase thickness). Two sublayers for THF and PBS and three sub-layers for acetone were used. with Respective a, c_0, and z_0 parameters used for fitting the data are listed in **Supporting Information Table S2.**

From the swollen ($h_{swollen}$) and dry (h_{dry}) film thicknesses, the swelling ratio (α) was calculated using **Equation 1**.

$$\alpha = \frac{h_{swollen}}{h_{dry}} \quad \mathbf{(1)}$$

High Resolution Mass Spectrometry (HR-MS). HR-MS analyses were conducted on a Xevo G2-S QTOF mass spectrometer coupled to the Acquity UPLC Class Binary Solvent Manager and BTN Sample Manager (Waters, Corporation, Milford, MA). Mass spectrometer detection was operated in positive ionization mode using direct injection with ESI source. Samples were diluted in MilliQ water acidified with 0.1% formic acid. The TOF mass spectra were acquired in the resolution mode over the range of m/z 50-1200. A mass accuracy better than 5 ppm was achieved using a leucine-encephalin solution as lock-mass infused continuously using the LockSpray source.

Water contact angle measurements: Water contact angles were measured using a DataPhysics OCA 35 contact angle measurement instrument. Measurements were done by adding a 5 µL droplet of DI water on silicon wafer. The contact angle was determined using the software SCA20 from DataPhysics.

The final values are reported as an average of 9 data points (three wafers per sample and three points per wafer) ± standard deviation.

Statistical Analysis: Statistical analysis was performed using Orgin Pro. The data presented are in the form of mean ± standard deviation of the mean. One-way analysis of variance (ANOVA) with a Tukey comparison posthoc test was performed to test the statistical differences among data sets and the statistical significance is reported as *p< 0.05, **p<0.01, ***p<0.001 and ns- not significant.

PROCEDURES

Peptide synthesis

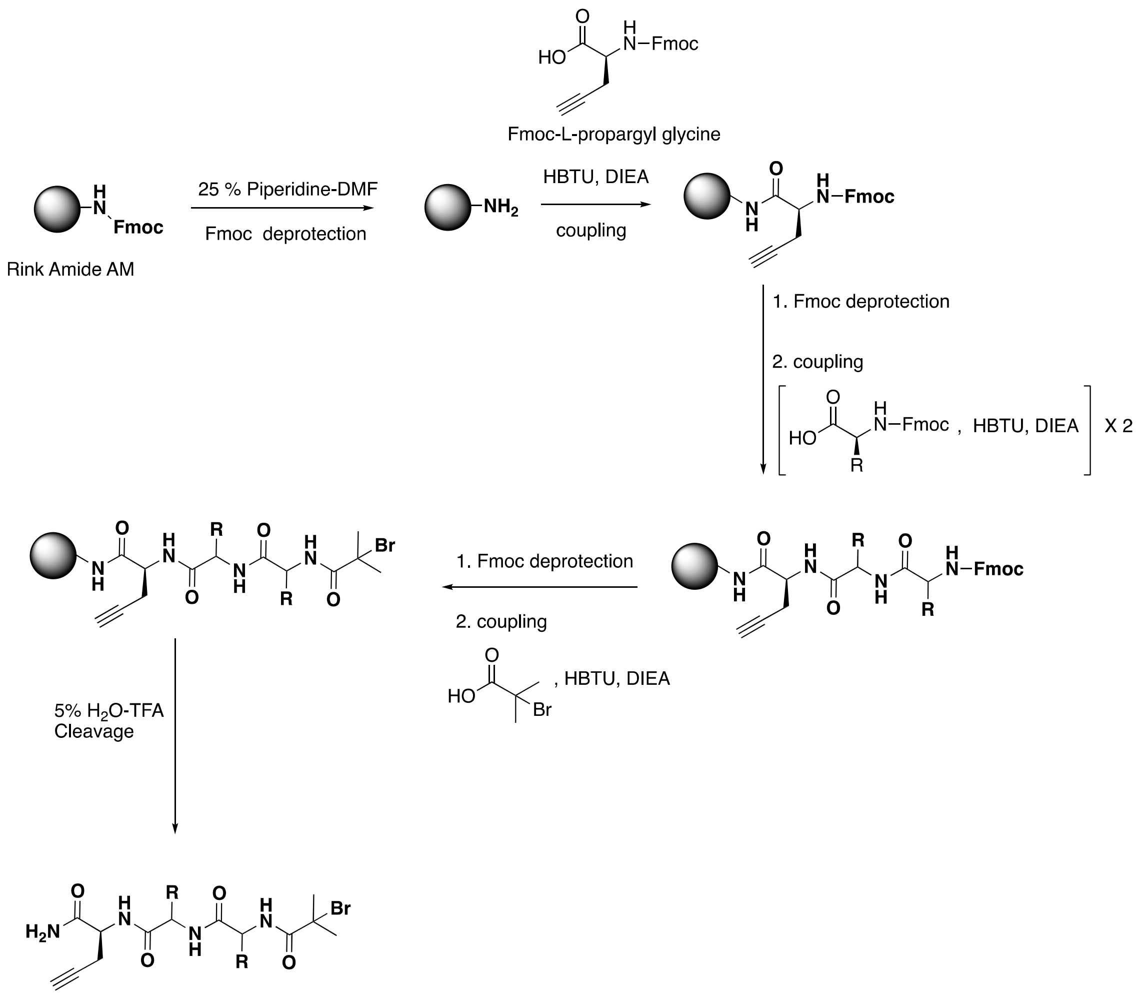

Peptides were synthesized using solid phase peptide synthesis via standard Fmoc chemistry. Briefly, Rink amide resin AM (420 mg, 0.294 mol) was swollen in 10 mL of a 1 : 1 (v/v) DMF : DCM mixture for 30 min. After draining the solvent, a Fmoc deprotection solution containing 25% (v/v) piperidine in DMF (5 mL) was added to the resin, and reaction was continued for 15 min, repeated twice. Between two steps, the solution was drained and the resin was rinsed with DMF (5 mL x 2) before adding the Fmoc deprotection solution for the second time. Following the deprotection reaction, the resin was washed with 10 mL DMF followed by 10 mL DCM, and a coupling cocktail containing 4 eq. of amino acid, 4 eq. of HBTU and 8 eq. of DIEA in DMF was added to the resin. The coupling reaction was allowed to contiune for 4 h followed by extensive washing of the resin with DMF and DCM. Subsequent

amino acid couplings were conducted by iterations of deprotection and coupling steps. After completion of the sequence, the peptide was cleaved by treating the resin with a cleavage cocktail (5 mL) consisting of 95% TFA, 2.5% MilliQ water and 2.5% TIPS (v/v) for 2 h. The cleaved peptide was collected in a flask, and the resin was repeatedly washed with TFA followed by DCM. After evaporating the mixture of TFA, DCM and TIPS, the remaining viscous mass was suspended in diethyl ether to precipitate the peptide. Finally, the peptide was isolated using centrifugation, and washed extensively with diethyl ether and dried under vacuum. The solid powder obtained from the previous step was re-dissolved either in MilliQ water (Pra-Gly-Gly-BiB and Pra-Ser-Ser-Bib), or in a mixture of 2% (v/v) acetonitrile (ACN) in MilliQ water (Pra-Phe-Phe-BiB), and loaded on a SPE cartridge (pre-soaked in 2% ACN-water). The peptide was purified by elution with with an ACN water mixture. The collected fractions were analyzed via ESI-MS, combined, and freeze dried to obtain solid white powder. **Supporting Information Table S3** lists for each of the synthesized peptides the specific elution conditions, and also summarizes the results from HR-MS analysis of the peptides. ESI MS spectra of the peptides are included in **Supporting Information Figures S4, S5 and S6.**

Synthesis of 3-(azidopropyl)triethoxysilane

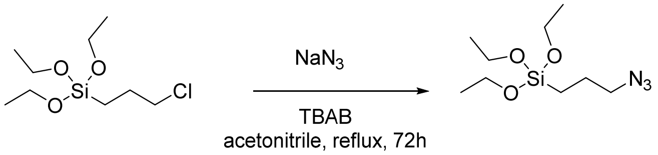

(3-Azidopropyl) triethoxysilane was synthesized according to a literature procedure.[3] A round-bottom flask was charged with NaN_3 (0.202 g, 3.0 mmol), TBAB (0.110 g, 0.4 mmol), and 7.5 mL dry acetonitrile. After the addition of (3-chloropropyl)triethoxysilane (0.500 mL, 2.0 mmol), the resulting mixture was heated to 80 °C for 48 hours. After that, the reaction was cooled, and diluted with diethyl ether and the resulting suspension was filtered. The filtrate was concentrated under reduced pressure to yield (3-azidopropyl)triethoxysilane as a clear oil (0.439 mL, 85%). ^1H NMR (400 MHz, $CDCl_3$) δ = 3.81 (q, 6H, J=6.94 Hz), 3.25 (t, 2H, J=6.80 Hz), 1.68 (m, 2H), 1.21 (t, 9H, J=6.9 Hz), 0.65 (m, 2H),

^{13}C NMR (400 MHz, $CDCl_3$) δ = 58.54, 53.92, 22.77, 18.37, 7.70. ^{1}H and ^{13}C NMR spectra are included in **Supporting Information Figures S7 and S8**.

Synthesis of 2-bromo-2-methyl-N-(3-(triethoxysilyl)-propyl)propanamide

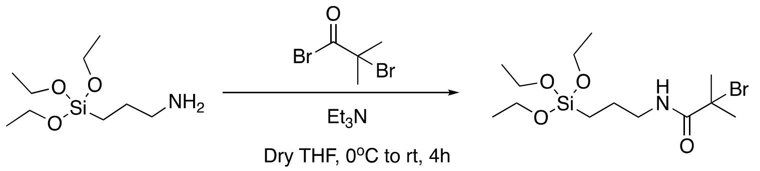

2-Bromo-2-methyl-*N*-(3-triethoxysilyl-propyl)-propionamide was synthesized according to a modified literature procedure.[4] 2-Bromoisobutrylbromide (BiBB) (2.18 mL (2.79 g, 17.6 mmol) was added dropwise to an ice-cooled (0 °C) solution of 2.95 mL (2.79 g, 18.90 mmol) (3-aminopropyl)triethoxysilane and 2.64 mL (1.31 g, 18.9 mmol) triethylamine in 20 mL dry THF. The solution was stirred for 3 hours, and the white precipitates were filtered off. The solution was concentrated under vacuum and the residue was redissolved in THF and passed through a silica column to yield 2-bromo-2-methyl-*N*-(3-triethoxysilyl-propyl)-propionamide (2.35 g, 56%) as a clear yellow oil. ^{1}H-NMR ($CDCl_3$, 400 MHz, ppm): δ = 6.85 (s br, 1H, NH), 3.83 (q, J= 7.06 Hz, 6H), 3.26 (q, J= 6.91 Hz, 2H), 1.93 (s, 6H), 1.65 (q, 2H), 1.21 (t, J= 6.89 Hz, 9H), 0.64 (t, J= 8.15 Hz, 2H). ^{13}C NMR (400 MHz, $CDCl_3$) δ = 171.99, 165.83, 63.40, 58.57, 42.69, 32.73, 30.11, 22.79, 18.41, 7.66. ^{1}H and ^{13}C-NMR spectra are included in **Supporting Information Figure S9 and S10**.

Synthesis of hex-5-en-1-yl 2-bromo-2-methylpropanoate

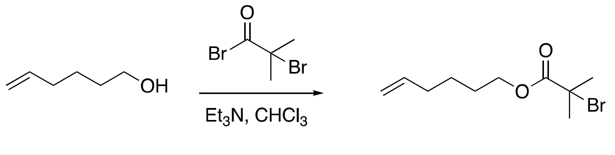

A dry flask equipped with a magnetic stirrer was charged with dry and degassed DCM (~30 mL), 5-hexen-1-ol (4.38 g), and dry triethylamine (4.35 g, 1 eq.).[5] The reaction solution was cooled to 0 °C, and α-bromoisobutyryl bromide (9.93 g, 1 eq.) was added to the solution dropwise. The reaction mixture was allowed to stir under an inert atmosphere at 0 °C for one hour, then at room temperature for another

3 hours. The precipitated salt was removed by filtration. The filtrate was washed with water 3 times, then dried over $MgSO_4$. The solvent was removed *in vacuo* to afford the product hex-5-en-1-yl 2-bromo-2-methylpropanoate as a yellowish oil and was directly taken into the next step (10.9 g, 94%). ^{1}H NMR (400 MHz, $CDCl_3$) δ = 5.80 (ddt, 1H, J = 16.9, 10.2, 6.6 Hz), 5.07 – 4.93 (m, 2H), 4.18 (t, 2H, J = 6.5 Hz), 2.15 – 2.04 (m, 2H), 1.93 (s, 6H), 1.70 (dq, 2H, J = 8.6, 6.4 Hz), 1.55 – 1.44 (m, 2H). ^{1}H spectrum is included in **Supporting Information Figure S11**.

Synthesis of 6-(chlorodimethylsilyl)hexyl 2-bromo-2-methylpropanoate

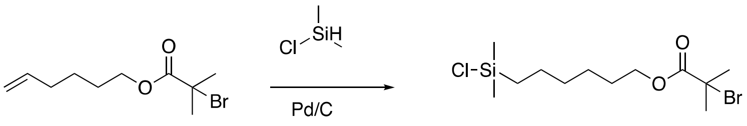

Dimethylchlorosilane (46 mL, 10 eq.), Pt/C (83 mg, 10%, 0.01 mol%) and hex-5-en-1-yl 2-bromo-2-methylpropanoate (10.5 g) were added to a flask equipped with a magnetic stirrer. The mixture was refluxed at 50 °C under an inert atmosphere overnight. The catalyst was removed by filtration over anhydrous $MgSO_4$. Excess, unreacted dimethylchlorosilane was evaporated under reduced pressure and the product was purified by vacuum distillation (at 140 °C). A colourless oil was isolated (9.7 g, 73%). ^{1}H NMR (400 MHz, $CDCl_3$) δ = 4.17 (t, 2H, J = 6.6 Hz), 1.93 (s, 6H), 1.67 (q, 2H, J = 6.8 Hz), 1.54 – 1.23 (m, 6H), 0.88 – 0.75 (m, 2H), 0.40 (s, 6H). ^{13}C NMR (101 MHz, $CDCl_3$-*d*) δ = 171.88, 66.21, 56.13, 32.59, 30.93, 28.36, 25.56, 23.00, 19.02, 1.81, 0.53. ^{1}H and ^{13}NMR spectra are included in **Supporting Information Figure S12 and S13**.

Synthesis of undec-10-yn-1-yl methanesulfonate

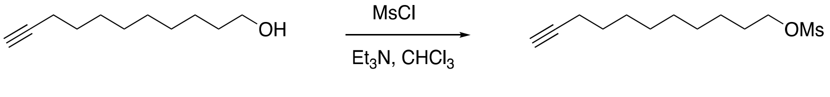

Undec-10-yn-1-yl methanesulfonate was synthesized according to a literature procedure.[6] To a stirred solution of undec-10-yn-1-ol (2.28 mL, 11.88 mmol) and triethylamine (1.82 mL, 13.07 mmol) in dry THF (60 mL) at 0 ºC, methanesulfonyl chloride (1.77 mL, 22.58 mmol) was added dropwise. (Note:

this reaction is exothermic and therefore was conducted in multiple small batches of under 3 mL). After stirring the reaction at room temperature for 2 hours, the THF layer was concentrated under vacuum and the resulting residue was dissolved in ethyl acetate and washed with 1N HCl and brine. Completion of the reaction was checked with TLC and stained with iodine (Rf = 0.9 in DCM) The organic layer was dried over sodium sulfate and concentrated under vacuum to yield pale yellow liquid (Crude yield = 1.97 g, 67 %). The product was taken to the next step without purification. ^{1}H-NMR ($CDCl_3$, 400 MHz, ppm): 4.20 (t, J= 6.27 Hz, 2H), 2.99 (s, 3H), 2.17 (dt, J= 2.64 Hz, 7.08 Hz, 2H), 1.92 (t, J= 2.64 Hz, 1H), 1.76-1.69 (m, 2H), 1.50-1.54 (m, 2H), 1.38 (m, 4H) 1.29 (m, 6H). ^{13}C NMR (400 MHz, $CDCl_3$) δ = 84.80, 70.28, 68.18, 37.46, 29.34, 29.21, 29.04, 28.74, 28.51, 25.47, 18.48. ^{1}H and ^{13}C-NMR spectra are included in **Supporting Information Figure S14 and S15**.

Synthesis of 11-azidoundec-1-yne

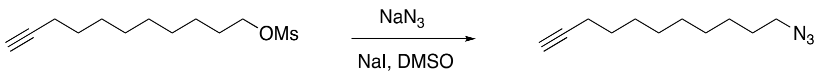

11-azidoundec-1-yne was synthesized according to a literature procedure.[6] To a stirred solution of undec-(10-yn-1-yl)methanesulfonate (1.45 g, 5.9 mmol) in 40 mL DMF, excess of sodium azide (1.91 g, 29.89 mmol) was added in a single portion. After refluxing for 4 hours, the reaction was cooled to room temperature, and 100 mL of a 1:1 mixture of water and hexanes were added. The organic layer was extracted and washed with a saturated aqueous solution of sodium bicarbonate (50 mL) and with brine (50 mL) and then dried over anhydrous sodium sulfate, filtered, and concentrated to yield a pale yellow liquid. The product was taken to the next step without purification (Crude yield 0.696 g, 88.7 %). ^{1}H-NMR ($CDCl_3$, 400 MHz, ppm): δ 3.23 (t, *J* = 7.0 Hz, 2H), 2.16 (td, *J* = 7.1, 2.6 Hz, 2H), 1.92 (t, *J* = 2.6 Hz, 1H), 1.62 – 1.45 (m, 4H), 1.28 (s, 11H), ^{13}C NMR (101 MHz, $CDCl_3$) δ 85.06, 68.50, 51.85, 29.71, 29.47, 29.37, 29.22, 29.07, 28.84, 27.08, 18.77. ^{1}H and ^{13}C-NMR spectra are included in **Supporting Information Figure S16 and S17**.

Synthesis of undec-10-yn-1-amine

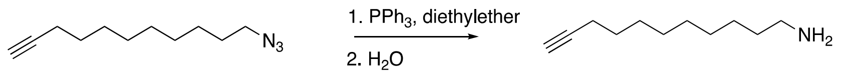

undec-10-yn-1-amine was synthesized according to a literature procedure.[7] To a stirred solution of 11-azidoundec-1-yne (0.696 g, 3.6 mmol) in diethylether (1.2 mL), triphenylphosphine (1.13 g, 4.32 mmol) was added in a single portion at 0 ºC. After stirring the reaction mixture for 15 hours, 1.2 mL water was added and the reaction allowed to stir to completion. Completion was monitored via TLC. After completion, the solvent was evaporated under vacuum and residue was dissolved in water. The aqueous layer was acidified to pH 2, and washed with ethyl acetate to remove the organic impurities like triphenylphosphine oxide (TPPO). Then the aqueous layer was basified to pH 9 and the product was extracted with ethyl acetate. The organic layers were combined, dried over sodium sulfate, and concentrated under vacuum to yield a white solid (0.423 g, 70 %). The product was taken to the next step without purification. ^{1}H NMR (400 MHz, $CDCl_3$) δ 2.68 (t, J= 7.04 Hz, 2H), 2.16 (dt, J= 2.68 Hz, 7.04 Hz, 2H), 1.92 (t, J= 2.67 Hz, 1H), 1.86 (bs, 2H), 1.51 (m, 2H), 1.44 (m, 2H), 1.38 (m, 2H), 1.28 (m, 8H) 9H). The ^{1}H spectrum is included in **Supporting Information Figure S18**.

Synthesis of 2-bromo-2-methyl-N-(undec-10-yn-1-yl) propenamide.

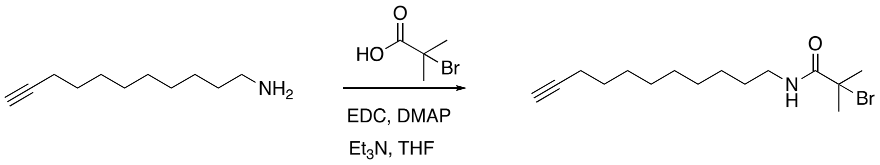

To a stirred solution of undec-10-yn-1-amine (200 mg, 1.2 mmol), 2-bromo-2-methyl propionic acid (219 mg, 1.32 mmol), and DMAP (189 mg, 1.55 mmol) in dry DCM (15 mL), EDC.HCl (363 mg, 1.55 mmol) was added in a single portion at 0 °C. The reaction was stirred at 0 °C for 30 min, and then at room temperature for 15 hours. The progress of the reaction was monitored via TLC. After completion, the reaction mixture was washed with 1N HCl followed by saturated sodium bicarbonate solution and

finally with brine. The organic layers were combined, dried over sodium sulfate, and concentrated under vacuum to yield colourless liquid. The final product was purified by passing through a silica column and eluting with pure DCM. The final product is obtained as a colorless liquid at room temperature, which solidifies at 4 ºC to a pale white solid (0.298 g, 70 %). ^{1}H NMR (400 MHz, $CDCl_3$) δ 6.71 (s, NH), 3.25 (td, J = 7.2, 5.8 Hz, 2H), 2.17 (td, J = 7.1, 2.7 Hz, 2H), 1.94 (s, 6H), 1.93 (t, 1H), 1.56 – 1.47 (m, 4H), 1.43 – 1.36 (m, 2H), 1.30 (m, 8H) 9H). ^{13}C NMR (101 MHz, $CDCl_3$) δ 172.30, 85.19, 68.54, 64.12, 40.87, 33.13, 30.15, 29.78, 29.72, 29.61, 29.44, 29.14, 28.91, 27.23, 18.84. ^{1}H and ^{13}C-NMR spectra are included in **Supporting Information Figure S19 and S20**.

Preparation of azide functionalized silicon wafers

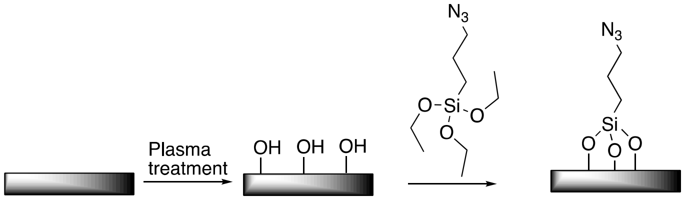

12 silicon wafers were put into a holder and placed inside a wide-mouthed Schlenk flask, and ultra-sonicated with ethanol, water, and acetone for 5 min, and then dried under vacuum for 1 h. Clean and dry wafers were then treated with oxygen plasma for 20 min. Meanwhile, 120 μL 3-(azidopropyl)triethoxysilane (0.477 mmol, density = 0.981 g/mL) was added into a dry round bottom flask under nitrogen folllowed by 15 mL dry toluene. The total reaction molarity was 31.8 mM. The mixture was swirled, and poured into the reaction flask (a wide-mouthed Schlenk tube) along with a small stir bar. The reaction flask was securely placed on a stir plate and the mixture was allowed to stir at 400 rpm under nitrogen flow. Plasma treated wafers were immediately immersed into the above prepared solution, and reaction was allowed to proceed under nitrogen for 24 h at room temperature. Finally, the wafers were washed with toluene and ethanol and dried under a flow of nitrogen and then under vacuum overnight.

Preparation of peptide-functionalized initiator monolayers

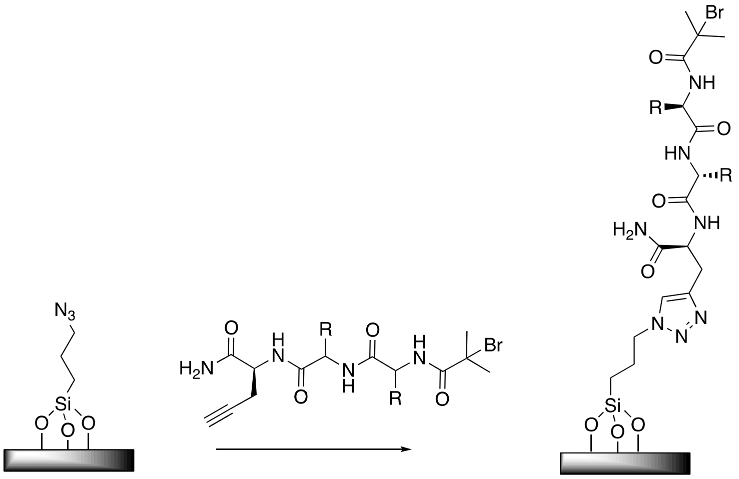

For the modification of azide-functionalized wafers with peptides, click reactions were performed in three separate vials containing different peptides, with 6 azide-modified silicon wafers in each vial. To prepare the peptide mixture, 58.97 mg TBTA (0.111 mmol) and 0.044 mmol peptide (16.68 mg Pra-Gly-Gly-BiB, 19.35 mg Pra-Ser-Ser-Bib or 24.69 mg Pra-Phe-Phe-Bib) were weighed out separately in a clean and dry Schlenk flask, and dissolved in 1.5 mL of DMSO. The flask was flushed with N_2 for 2 min, and then subjected to three freeze-pump-thaw cycles. After that, the reaction mixture was frozen, and 15.95 mg CuBr (0.111 mmol) was added to the reaction mixture. The solution was allowed to thaw and the contents were dissolved. Following another freeze-pump-thaw cycle, the solution was transferred into three individual vials containing 6 azide-modified silicon wafers. The vials were sealed under nitrogen and heated to 60°C for 24 h. After 24 h, the wafers were removed from the solution, washed extensively with DMSO and ethanol and then dried under vacuum.

Preparation of 2-bromo-2-methyl-*N*-(3-triethoxysilyl-propyl)-propionamide-functionalized initiator monolayers

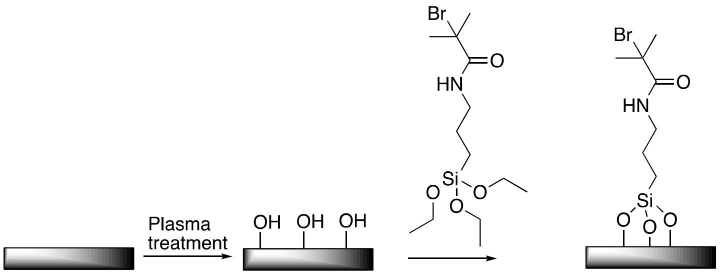

12 silicon wafers were put into a holder and placed inside a wide-mouthed Schlenk flask and ultra-sonicated with ethanol, water, and acetone for 5 min, and then dried under vacuum for 1 h. Clean and dry wafers were then treated with oxygen plasma for 20 min. Meanwhile, the reaction solution was prepared as follows: 100 μL 2-bromo-2-methyl-*N*-(3-triethoxysilyl-propyl)-propionamide) (0.324 mmol, density = 1.2 g/mL) was added into a dry round bottom flask under nitrogen and 20 mL dry toluene was added. Total reaction molarity was around 16.2 mM. The mixture was swirled, and poured into the reaction flask (a wide-mouthed Schlenk tube) along with a small stir bar. The reaction flask was securely placed on a stir plate and the reaction mixture was allowed to swirl under nitrogen flow. Plasma treated wafers were immediately immersed into the above prepared solution and reaction was continued under nitrogen for 24 h at room temperature. Finally, the wafers were washed with toluene and ethanol and dried under a flow of nitrogen and then under vacuum overnight.

Preparation of 6-(chlorodimethylsilyl)hexyl 2-bromo-2-methylpropanoate-functionalized initiator monolayers

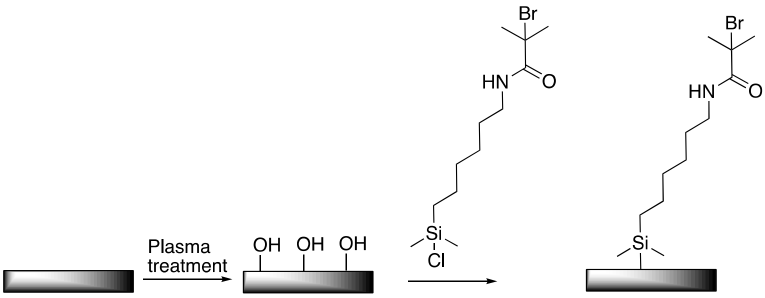

12 Silicon wafers were put into a holder and placed inside a wide-mouthed Schlenk flask and ultra-sonicated with ethanol, water, and acetone for 5 min, and then dried under vacuum for 1 h. Clean and dry wafers were then treated with oxygen plasma for 20 min. Meanwhile, the reaction solution was prepared as follows: 165 μL of 6-(chlorodimethylsilyl)hexyl 2-bromo-2-methylpropanoate (0.416

mmol, density = 0.867 g/mL) was added into a dry round bottom flask under nitrogen and 30 mL dry toluene was added. The mixture was swirled, and poured into the reaction flask (a wide-mouthed Schlenk tube) along with a small stir bar. The reaction flask was securely placed on a stir plate and the reaction mixture was allowed to swirl under nitrogen flow. Plasma treated wafers were immediately immersed into the above prepared solution and reaction was continued under nitrogen for 24 h at room temperature. Finally, the wafers were washed with toluene and ethanol and dried under a flow of nitrogen and then under vacuum overnight.

Preparation of 2-bromo-2-methyl-*N*-(undec-10-yn-1-yl) propenamide-functionalized initiator monolayers

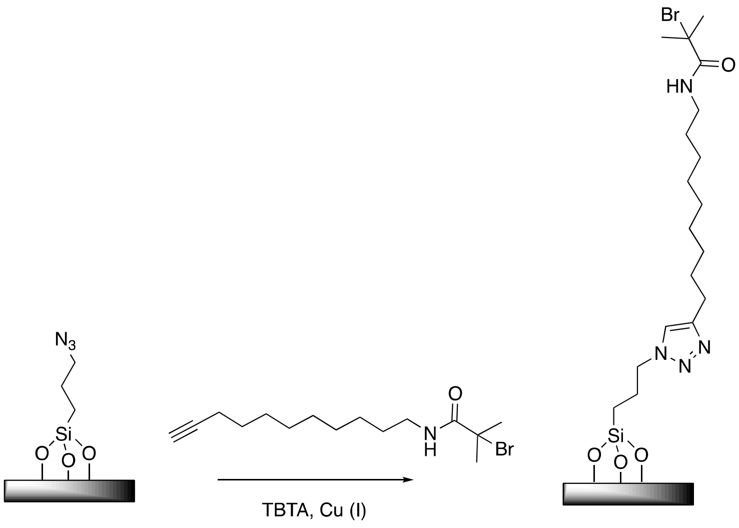

Similar to the preparation of peptide-functionalized initiator monolayers, 2-bromo-2-methyl-*N*-(undec-10-yn-1-yl) propenamide-funtionalized monolayers were synthesized using click chemistry. 58.97 mg TBTA (0.111 mmol) and 0.044 mmol (14.06 mg) 2-bromo-2-methyl-N-(undec-10-yn-1-yl) propenamide were weighed in a clean and dry Schlenk flask and dissolved in 1.5 mL DMSO. The flask was flushed with N_2 for 2 min, and then subjected to three freeze-pump-thaw cycles. After that, the reaction mixture was frozen, and 15.95 mg CuBr (0.111 mmol) was added to the reaction mixture. The solution was allowed to thaw and the contents were dissolved. Following another freeze-pump-thaw cycle, the solution was transferred into vial containing 6 azide-modified silicon wafers. The vial was

sealed under nitrogen and heated to 60 °C for 24 h. After 24 h, the wafers were removed from the solution, washed extensively with DMSO and ethanol and then dried under vacuum.

Surface-initiated atom transfer radical polymerization of tBMA

11.3 mL t-butyl methacrylate (70.32 mmol) was added to a clean and dry Schlenk tube followed by 8 mL DMSO (reagent grade > 99.9%). The mixture was subjected to three freeze-pump-thaw cycles, after which 18.6 mg ascorbic acid (0.105 mmol) was added. Meanwhile, 2 mL DMSO was degassed in a 5 mL round-bottom flask for 15 min, before addition of 3.15 mg Cu(II) bromide (0.0146 mmol) and 30 μL PMDETA (0.1406 mmol). The resulting light-blue mixture was degassed for additional 30 min, and then transferred to the freshly freeze-pump-thawed mixture. The final colorless transparent solution (4 mL) was added to a vial containing 6 peptide-modified wafers or alkyl-initiator-modified wafers, and the vial was sealed with a septum and Parafilm. Each vial contained only one type of activated wafers to avoid errors. The final monomer : ligand : ascorbic acid : Cu(II) ratio was 10000 : 20 : 15 : 2. The polymerization reaction was allowed to continue for 4-6 h at room temperature. The reaction was quenched by adding methanol, and the wafers were washed extensively with DMSO, DCM, and acetone, and then dried under air and stored under vacuum until further analysis.

Surface initiated atom transfer radical polymerization of DMAEMA

8 mL DMAEMA (40 mmol), 5 mg Cu(II)Br (0.02 mmol), and 200 mg 2,2'-bipyridine (1 mmol) were added to a clean and dry Schlenk tube and dissolved in a (1:1) mixture of methanol and DI water (14 mL). After sonicating the mixture (yellow-golden in color) for 5 min, it was subjected to three freeze-pump-thaw cycles, and 40 mg Cu(I)Cl (0.4 mmol) was added under nitrogen. The final mixture with monomer : ligand : Cu(I) : Cu(II) ratio of 2000 : 50 : 20 : 1 was sonicated for 10 min after which it was transferred to vial (4 mL) containing 6 peptide-based ATRP initiator modified wafers under nitrogen and the vial was sealed with a septum and parafilm. The polymerization reaction was allowed to continue 6-8 h at room temperature. The reaction was quenched by adding methanol and the wafers were washed extensively with water and ethanol, and then dried under air and stored under vacuum until further analysis.

SUPPORTING SCHEMES

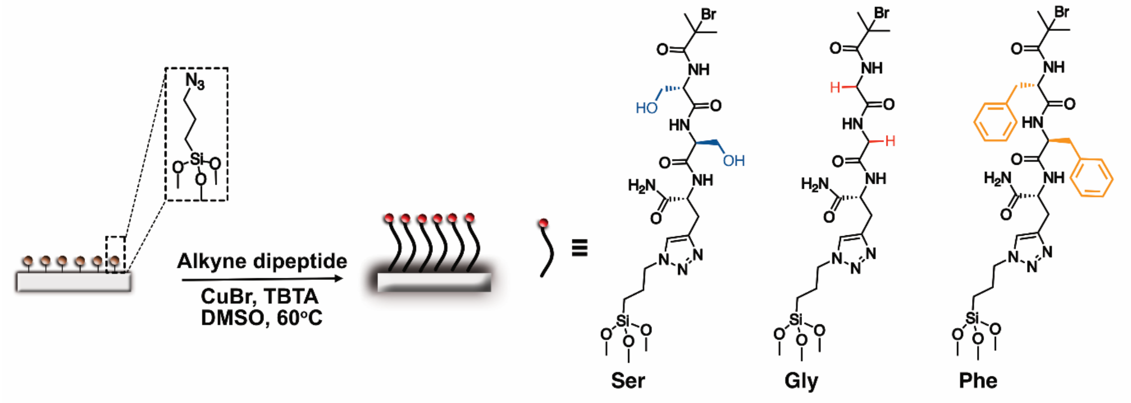

Scheme S1. Functionalization of silicon substrates with alkyne-functionalized diserine (**Ser**), diglycine (**Gly**) and diphenylalanine (**Phe**) peptide-based ATRP initiators via Cu-mediated click chemistry.

SUPPORTING TABLES

Table S1: Grafting densities and reduced grafting densities of the brushes, and number-average molecular weights (M_n) and radii of gyration (R_g) of the polymer tethers determined from analysis of the dry and swollen film thicknesses.

**Sample**	Dry film thickness (h_{dry}) (nm)	Swollen film thickness ($h_{swollen}$) (nm)[a]	M_n (kDa)[b]	Grafting density (σ) (chains / nm^2)[c]	R_g (nm)[d]	Reduced grafting density Σ (chains)[e]
**Ser-PtBMA**	104 ± 9	491 ± 23	337 ± 26	0.192 ± 0.030	12.9 ± 0.5	100 ± 10
**Gly-PtBMA**	122 ± 13	476 ± 15	297 ± 16	0.253 ± 0.035	12.1 ± 0.3	117 ± 12
**Phe-PtBMA**	161 ± 37	477 ± 16	263 ± 24	0.387 ± 0.119	11.4 ± 0.5	154 ± 34
**C3-PtBMA**	167 ± 28	474 ± 7	254 ± 24	0.414 ± 0.101	11.2 ± 0.5	160 ± 25
**C9-PtBMA**	155 ± 19	462 ± 8	253 ± 14	0.379 ± 0.061	11.2 ± 0.3	148 ± 16
**Ser-PDMAEMA**	109 ± 3	523 ± 14	987 ± 42	0.070 ± 0.003	21.0 ± 0.4	97 ± 3
**Gly-PDMAEMA**	130 ± 45	536 ± 83	949 ± 150	0.089 ± 0.033	20.6 ± 1.7	116 ± 41
**Phe-PDMAEMA**	66 ± 28	388 ± 105	815 ± 160	0.050 ± 0.014	19.0 ± 1.9	59 ± 25
**C3-PDMAEMA**	143 ± 39	557 ± 107	953 ± 182	0.096 ± 0.022	20.6 ± 2.1	127 ± 32
**C9-PDMAEMA**	134 ± 35	417 ± 112	636 ± 188	0.138 ± 0.031	16.7 ± 2.7	119 ± 28

a) Swollen film thicknesses of PtBMA brushes were measured in THF, and swollen brush thicknesses for PDMAEMA brushes in 10 mM PBS.
b) $M_n = N \times M_o$, where N (degree of polymerization) was estimated from Milner-Cates Model, with $M_{o(tBMA)}$ = 142.20 g/mol and $M_{o(DMAEMA)}$ = 157.21 g/mol.[8,9] Kuhn length (l_k) used to estimate N is calculated from $l_k = 2l_p$, where l_p is the persistence length. See Lodge and Hiemenz, *Polymer Chemistry*, 3rd edition (CRC Press, **2020**), Section 6.4, equation 6.4.6. l_p = 1.9 nm for PtBMA[10] and 0.8 nm PDMAEMA[11]. Excluded volume parameter for PtBMA and PDMAEMA is assumed to be 8 Å^3.
c) Grafting density estimated as $\sigma = (h_{dry}\, \rho\, N_A)/M_n$, where ρ is the polymer density and N_A Avogadro's number.
d) R_g is calculated from $R_g^2 = (N \times b^2)/6$ with b = 6.5 Å. See also Lodge and Hiemenz, *Polymer Chemistry*, 3rd edition (CRC Press, **2020**), Section 6.5. The value of b is the value for poly(methyl methacrylate) taken from Table 6.1, page 243 of that book.
d) Calculated from: $\Sigma = \sigma \pi R_g^2$
e) The reported errors represent the standard deviation from the average over the 6 – 9 individual samples that were evaluated.

Table S2. Parameters used for fitting swollen thicknesses of PtBMA and PDMAEAM brushes in various solvents. Refractive indices for ambient phase and substrate were provided by SEA v1.6.1 software.

#	layer	Thickness (nm)	n	a	c_0	$\mathbf{z_0}$
**Ambient**	air	∞	1.0003	-	-	-
	water		1.3316	-	-	-
	THF		1.4073	-	-	-
	acetone		1.3586	-	-	
**Polymer**	PtBMA (THF)	-	-	-0.628	1.115	0.044
	PtBMA (acetone)	-	-	-1.860	1.014	0.000
	PDMAEMA (PBS)	-	-	-0.889	0.921	0.221
**Substrate**	SiO_2	1.88	1.457	-	-	
	Si	∞	3.881	-	-	

Table S3. Details of peptide purification and MS analysis.

Peptide	**Purification details** (all percentages are v/v)	**Yield (%)**	**HRMS (ESI/QTOF)**	
			**Expected**	**Found**
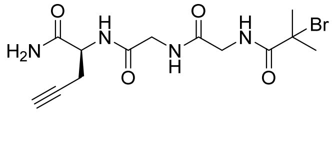 Pra-Gly-Gly-BiB	1. 2 % ACN-MilliQ water 5 mL<br>2. 30% ACN-MilliQ water 10 mL<br>3. 48% ACN-MilliQ water 5 mL	86 mg (78 %)	m/z: $[M + Na]^+$ Calculated for $C_{13}H_{19}BrN_4NaO_4^+$ 397.0482	397.0484
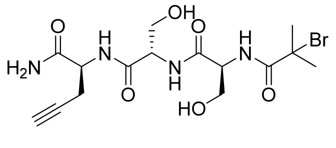 Pra-Ser-Ser-BiB	1. 2 % ACN-MilliQ water 5 mL<br>2. 48 % ACN-MilliQ water 10 mL	112.7 mg (88 %)	m/z: $[M + Na]^+$ Calculated for $C_{15}H_{23}BrN_4NaO_6^+$ 457.0693	457.0688
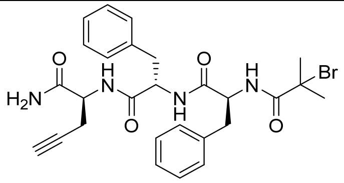 Pra-Phe-Phe-BiB	1. 2 % ACN-MilliQ water 5 mL<br>2. 20 % ACN-MilliQ water 10 mL	120 mg (73.4 %)	m/z: $[M + Na]^+$ Calculated for $C_{27}H_{31}BrN_4NaO_4^+$ 577.1421	577.1425

SUPPORTING FIGURES

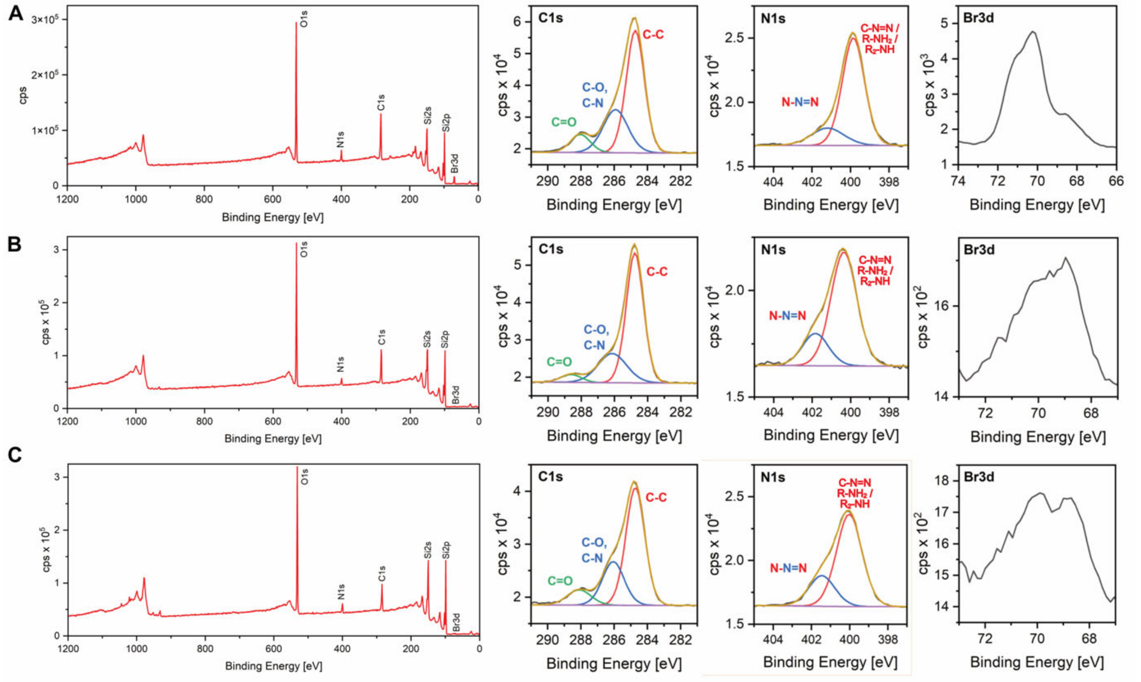

Figure S1: XPS survey spectra and C1s, N1s and Br3rd high resolution spectra for **A) Ser**, **B) Gly**, **and C) Phe**-modified silicon wafers.

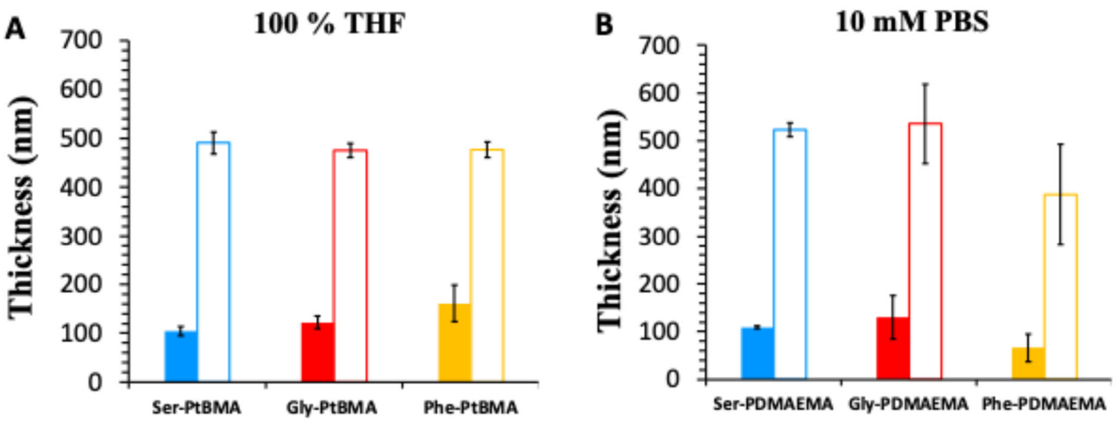

Figure S2: Dry (solid bars) and swollen (hollow bars) film thicknesses of **A)** PtBMA and **B)** PDMAEMA brushes grown from the different peptide-spacer modified ATRP inititators. Swollen film thicknesses of PtBMA brushes were measured in dry THF, and swollen film thicknesses of the PDMAEMA brushes in 10 mM PBS.

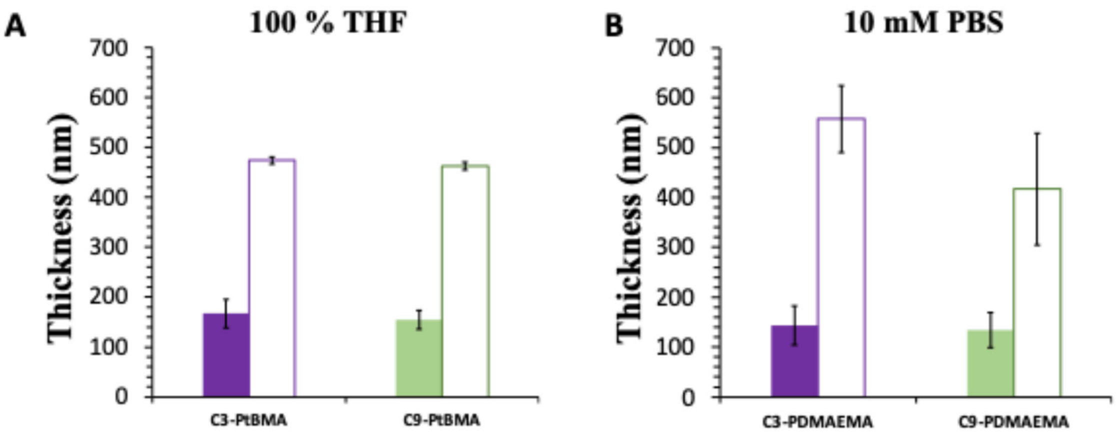

Figure S3: Dry (solid bars) and swollen (hollow bars) film thicknesses of **A)** PtBMA and **B)** PDMAEMA brushes grown from the different alkyl ATRP initiators. Swollen film thicknesses of PtBMA brushes were measured in dry THF, and swollen film thicknesses of the PDMAEMA brushes in 10 mM PBS

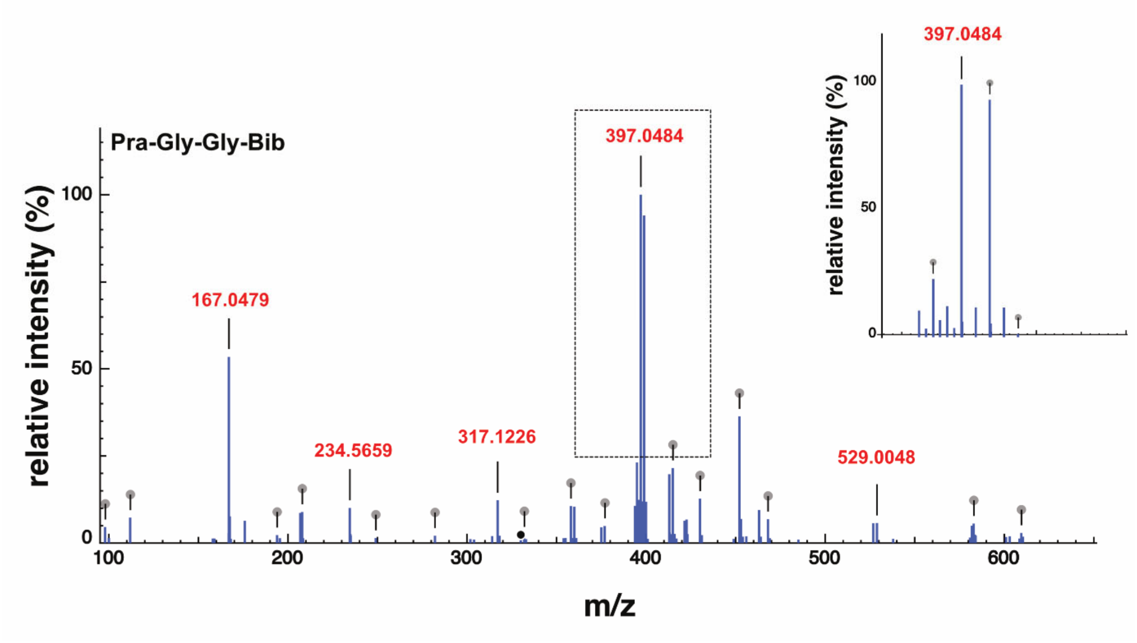

Figure S4: ESI/QTOF spectrum for Pra-Gly-Gly-Bib peptide: m/z: $[M+Na]^+$ Calculated for $C_{13}H_{19}BrN_4O_4^+$ 397.0482, found 397.0484. Inset shows the bromo pattern.

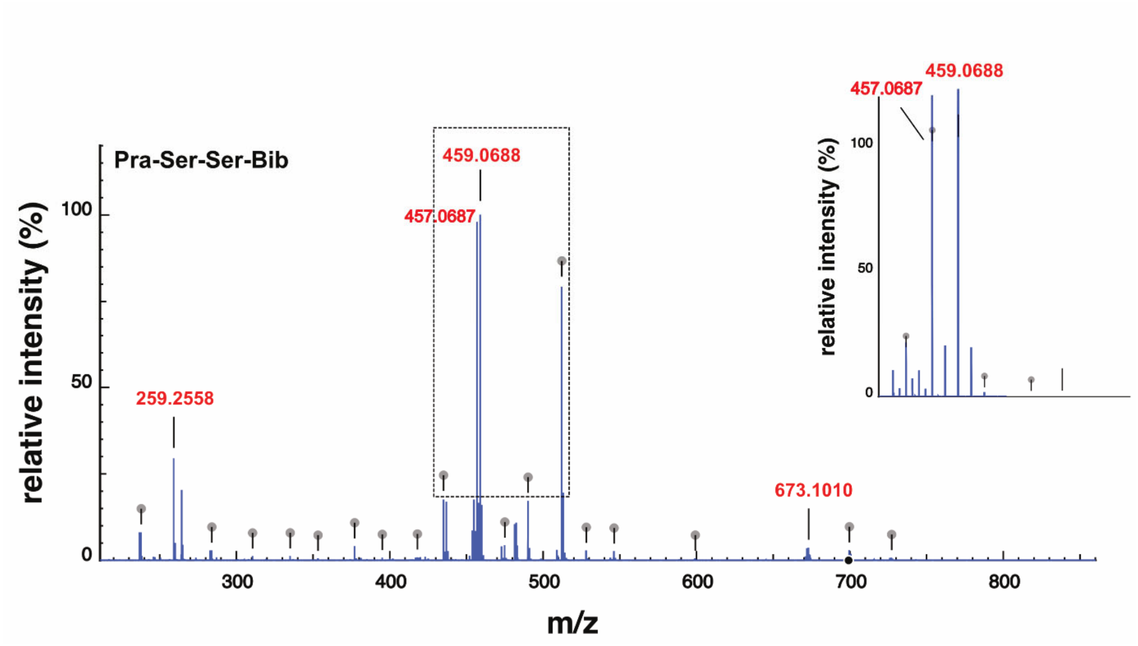

Figure S5: ESI/QTOF spectrum for Pra-Ser-Ser-Bib peptide: m/z: $[M + Na]^+$ Calcd for $C_{15}H_{23}BrN_4NaO_6^+$ 457.0693, found 457.0687. Inset shows the bromo pattern.

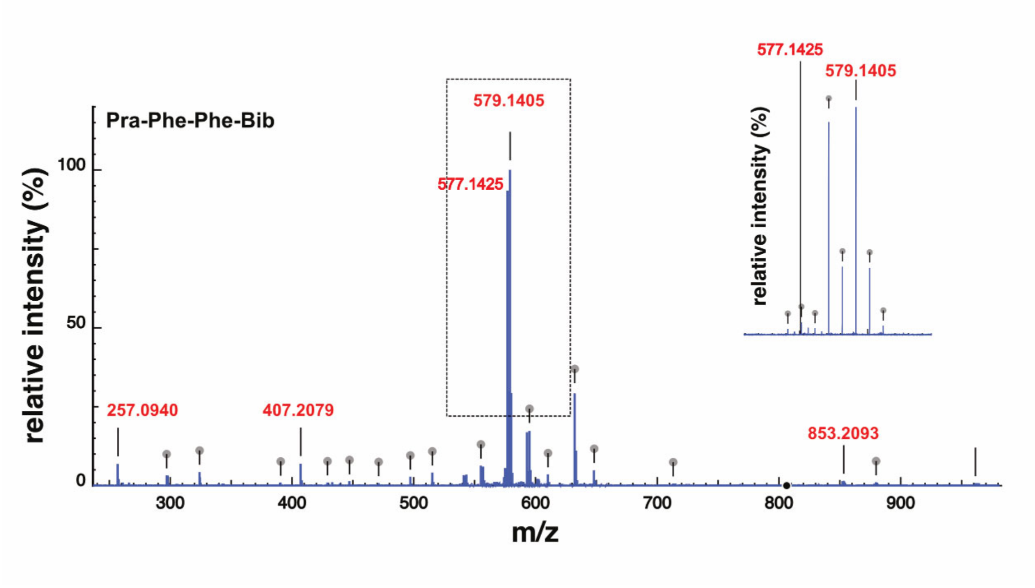

Figure S6: ESI/QTOF spectrum for Pra-Phe-Phe-Bib peptide: m/z: $[M + Na]^+$ Calcd for $C_{27}H_{31}BrN_4NaO_4^+$ 577.1421, found 577.1425. Inset shows the bromo pattern.

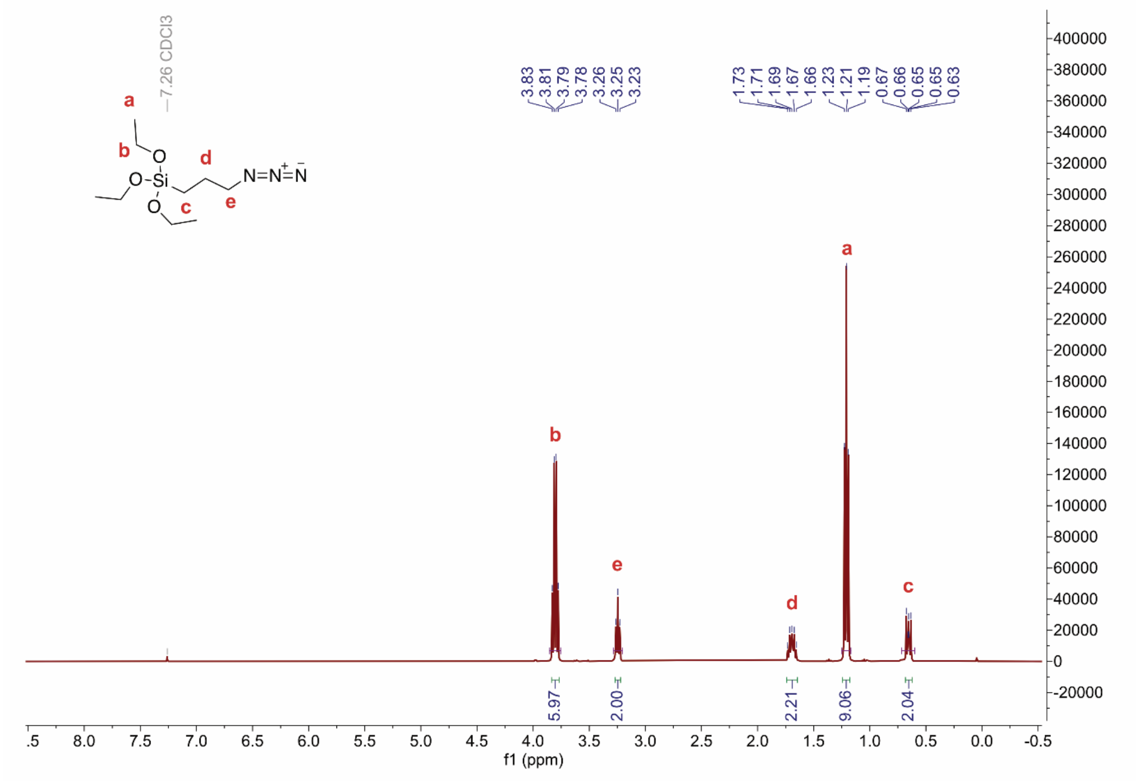

Figure S7: ^{1}H NMR spectrum of 3-(azidopropyl)triethoxy silane ($CDCl_3$).

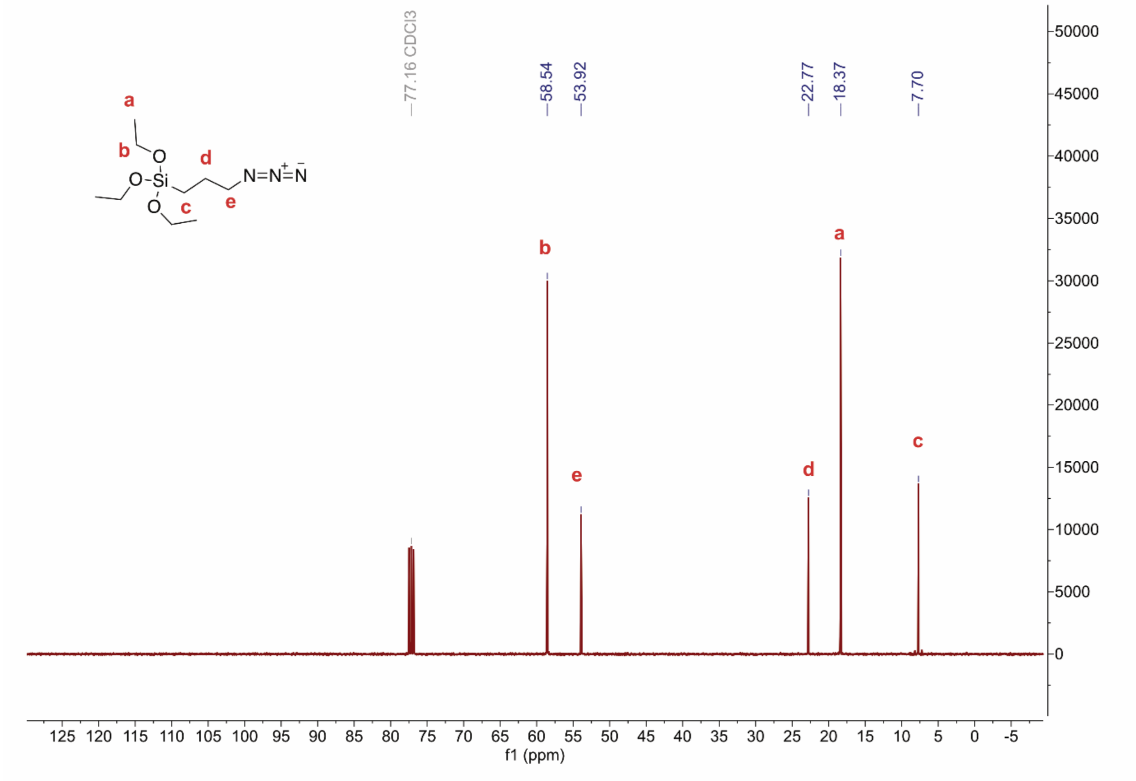

Figure S8: ^{13}C NMR spectrum of 3-(azidopropyl)triethoxysilane ($CDCl_3$).

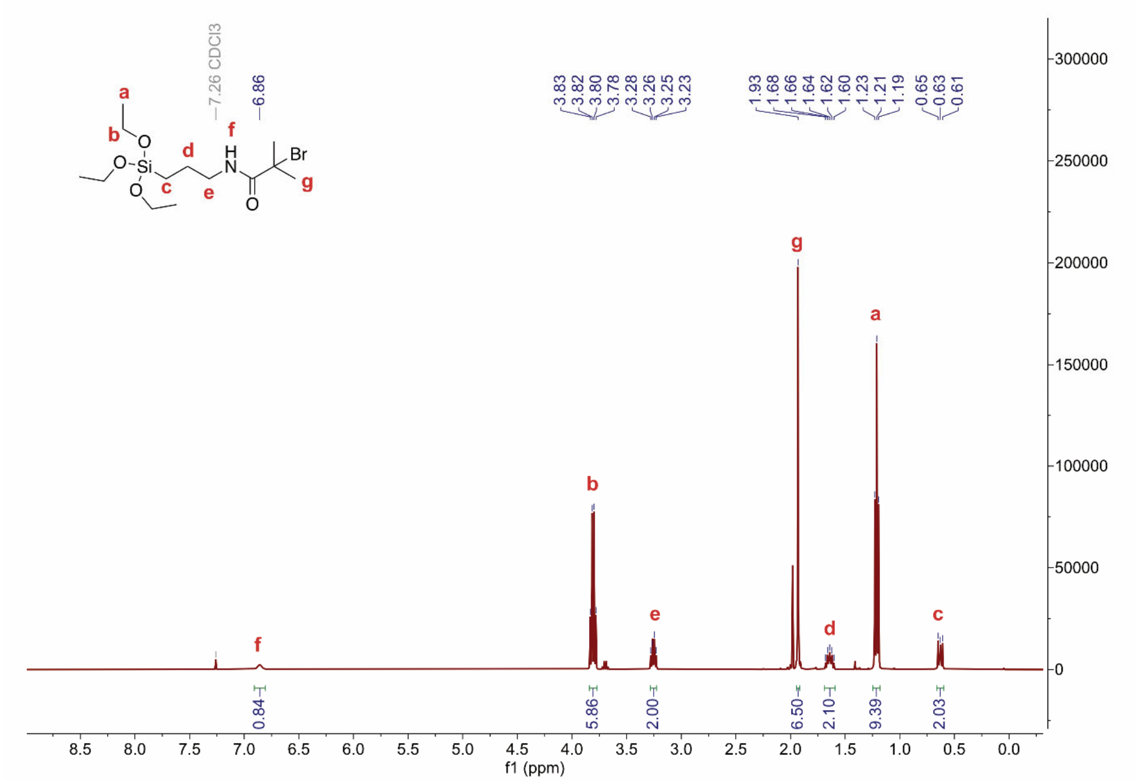

Figure S9: ^{1}H NMR spectrum of 2-bromo-2-methyl-*N*-(3-triethoxysilyl-propyl)-propionamide ($CDCl_3$).

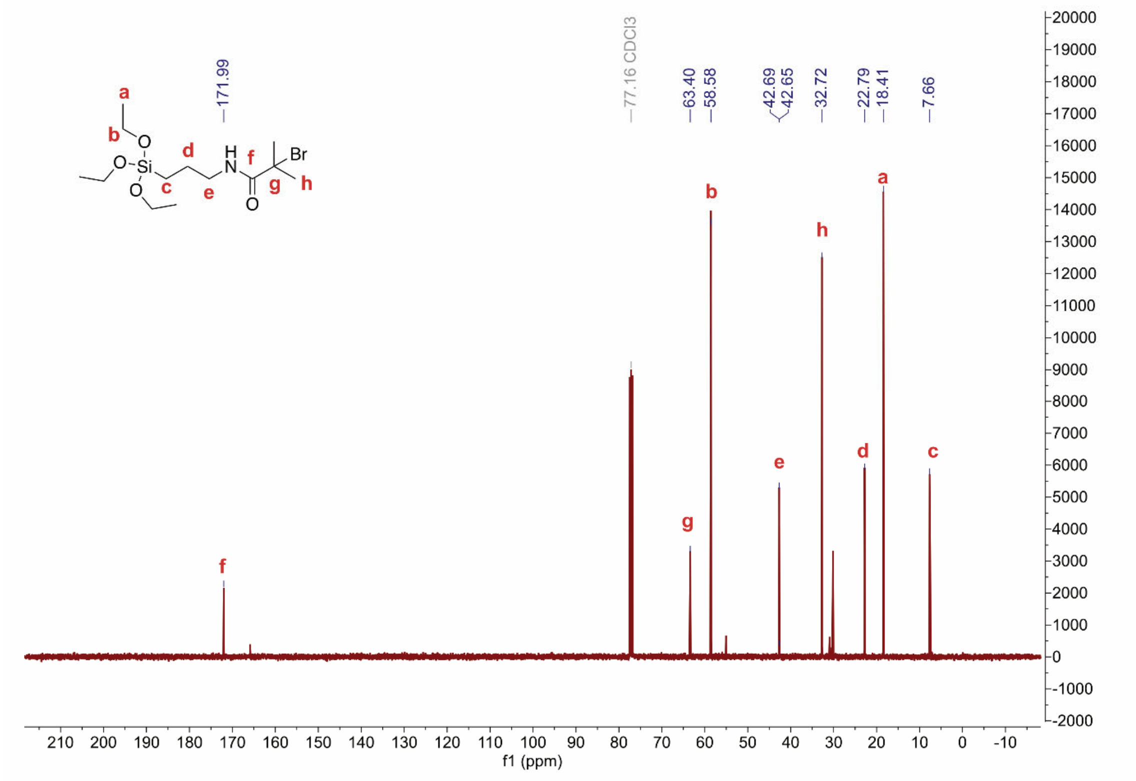

Figure S10: ^{13}C NMR spectrum of 2-bromo-2-methyl-*N*-(3-triethoxysilyl-propyl)-propionamide ($CDCl_3$).

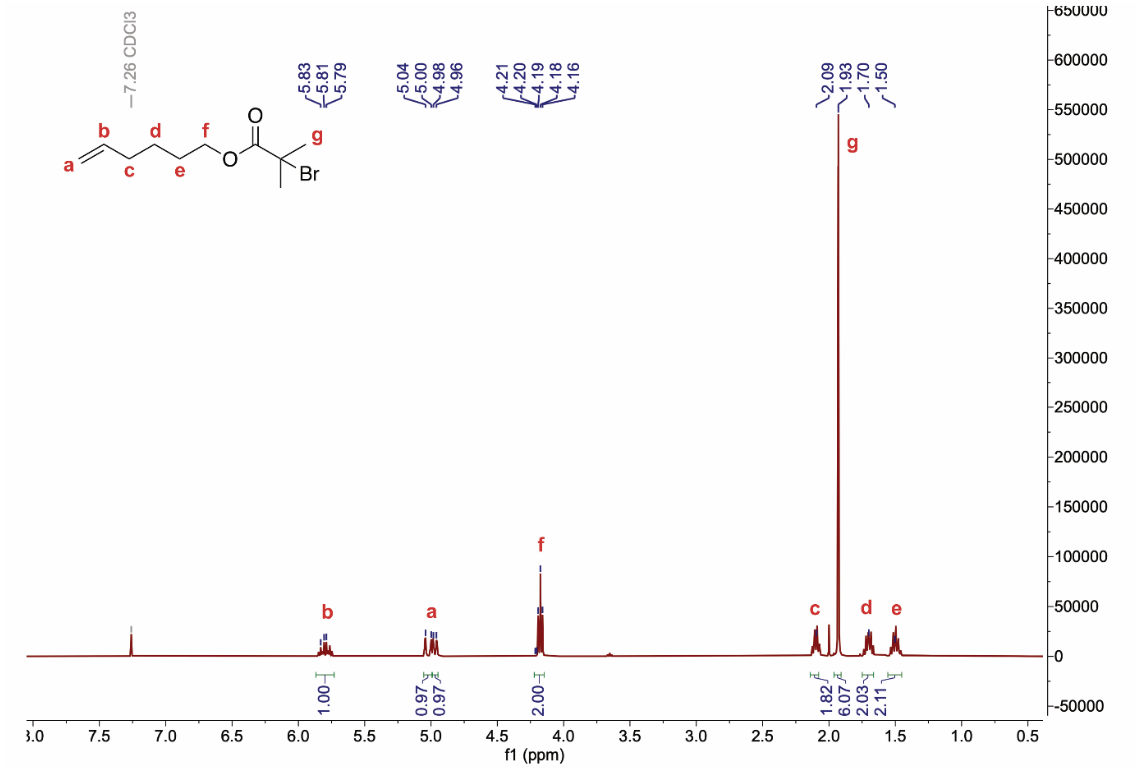

Figure S11: ^{1}H NMR spectrum of hex-5-en-1-yl 2-bromo-2-methylpropanoate ($CDCl_3$).

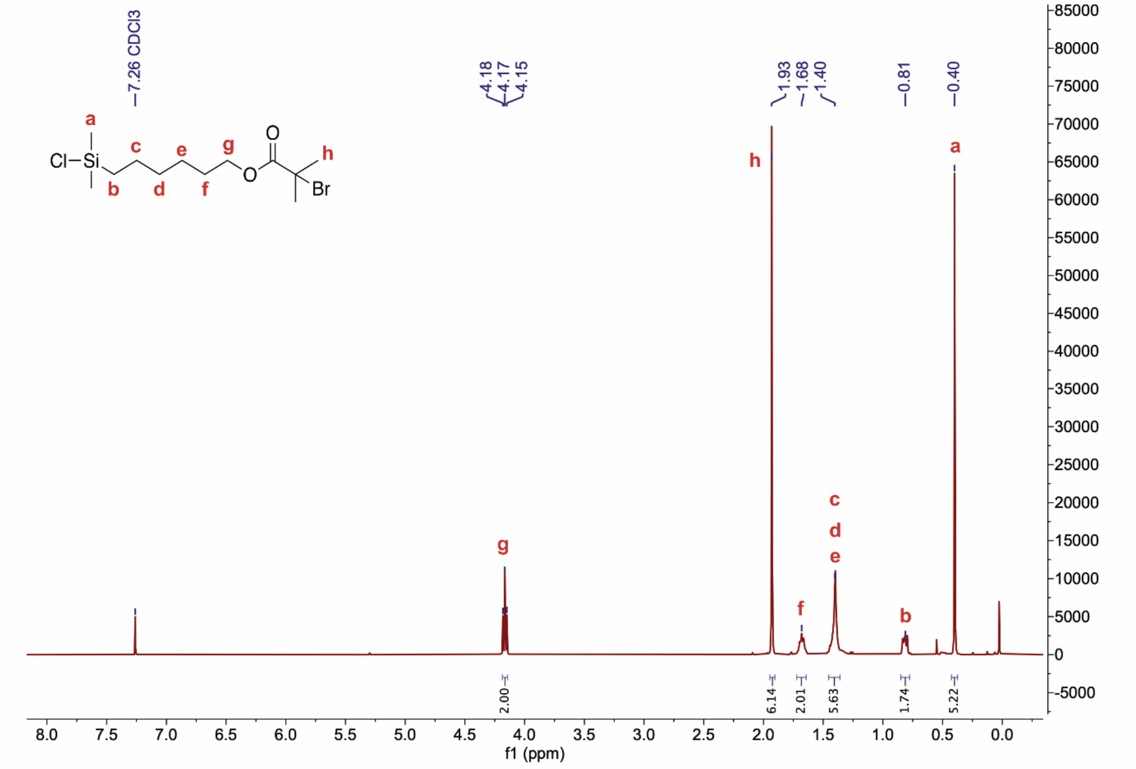

Figure S12: ^{1}H NMR spectrum of 6-(chlorodimethylsilyl)hexyl 2-bromo-2-methylpropanoate ($CDCl_3$).

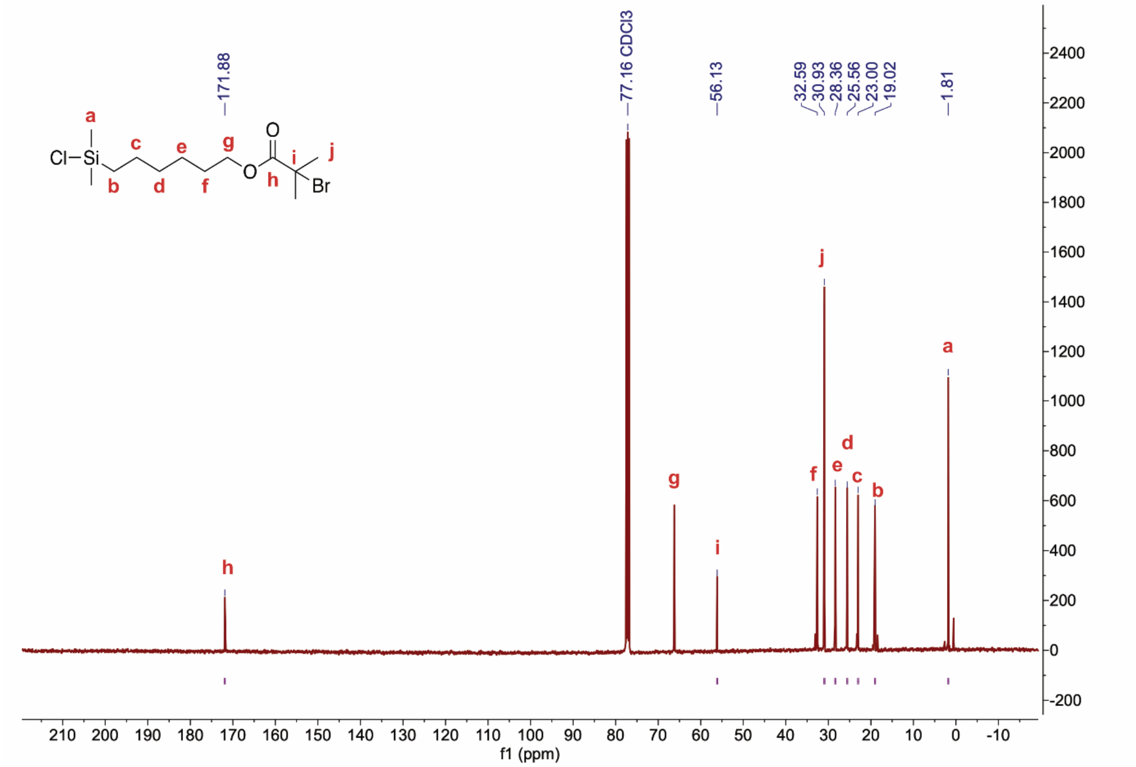

Figure S13: ^{13}C NMR spectrum of 6-(chlorodimethylsilyl)hexyl 2-bromo-2-methylpropanoate ($CDCl_3$).

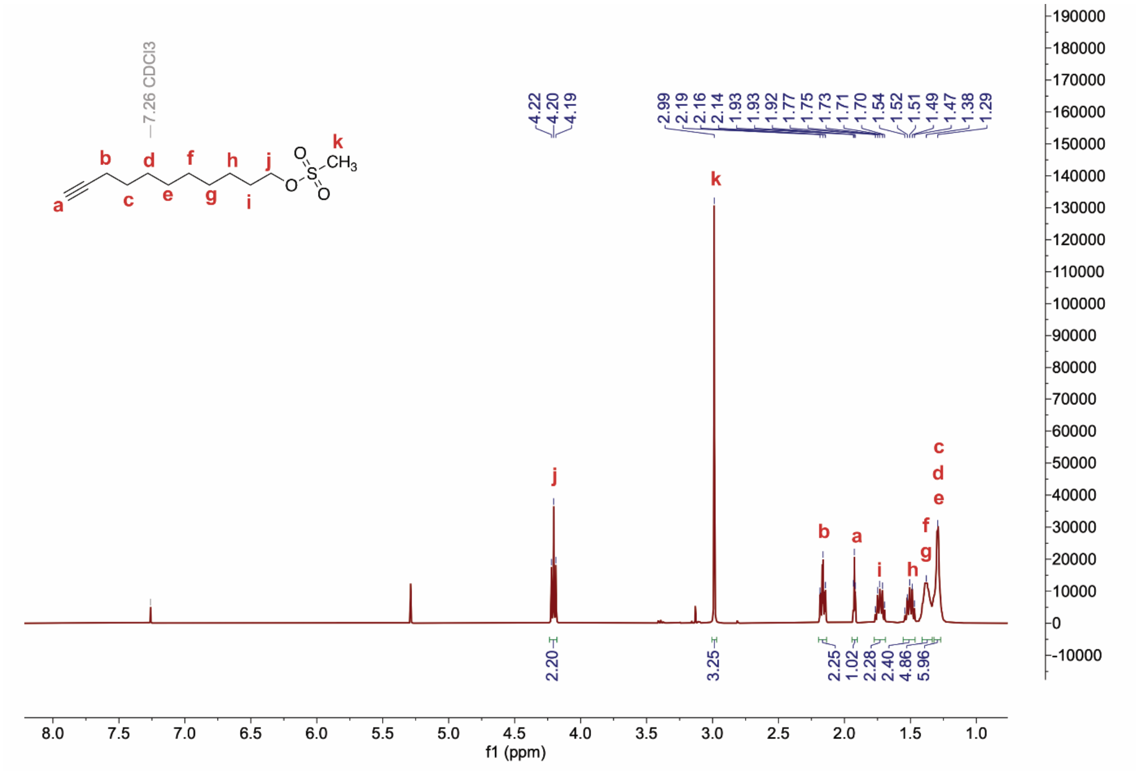

Figure S14: ^{1}H NMR spectrum of undec-10-yn-1-yl methanesulfonate ($CDCl_3$).

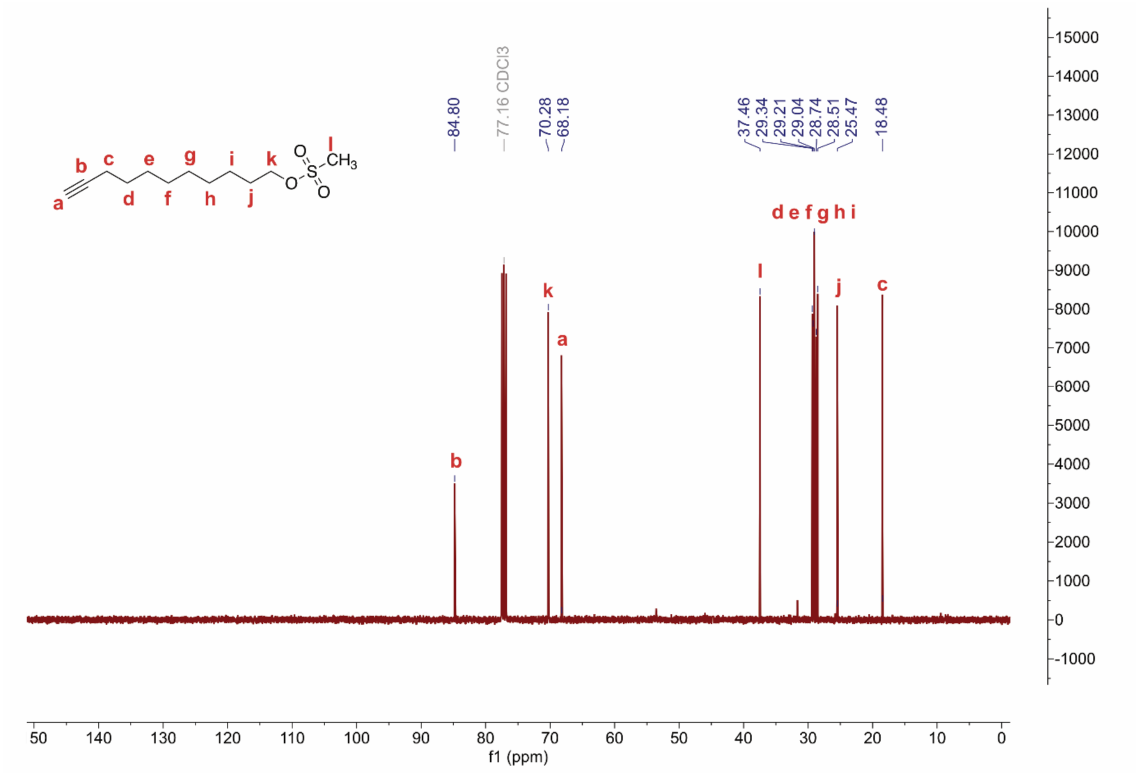

Figure S15: ^{13}C NMR spectrum of undec-10-yn-1-yl methanesulfonate ($CDCl_3$).

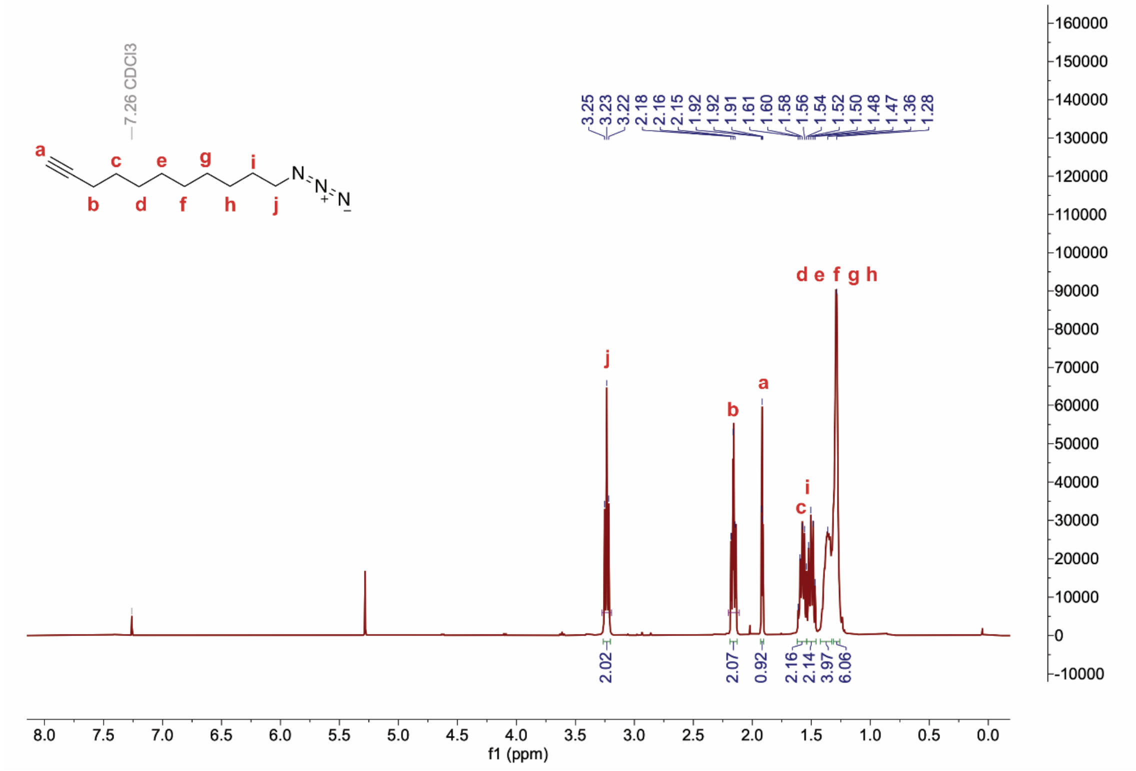

Figure S16: ^{1}H NMR spectrum of 11-azidoundec-1-yne ($CDCl_3$).

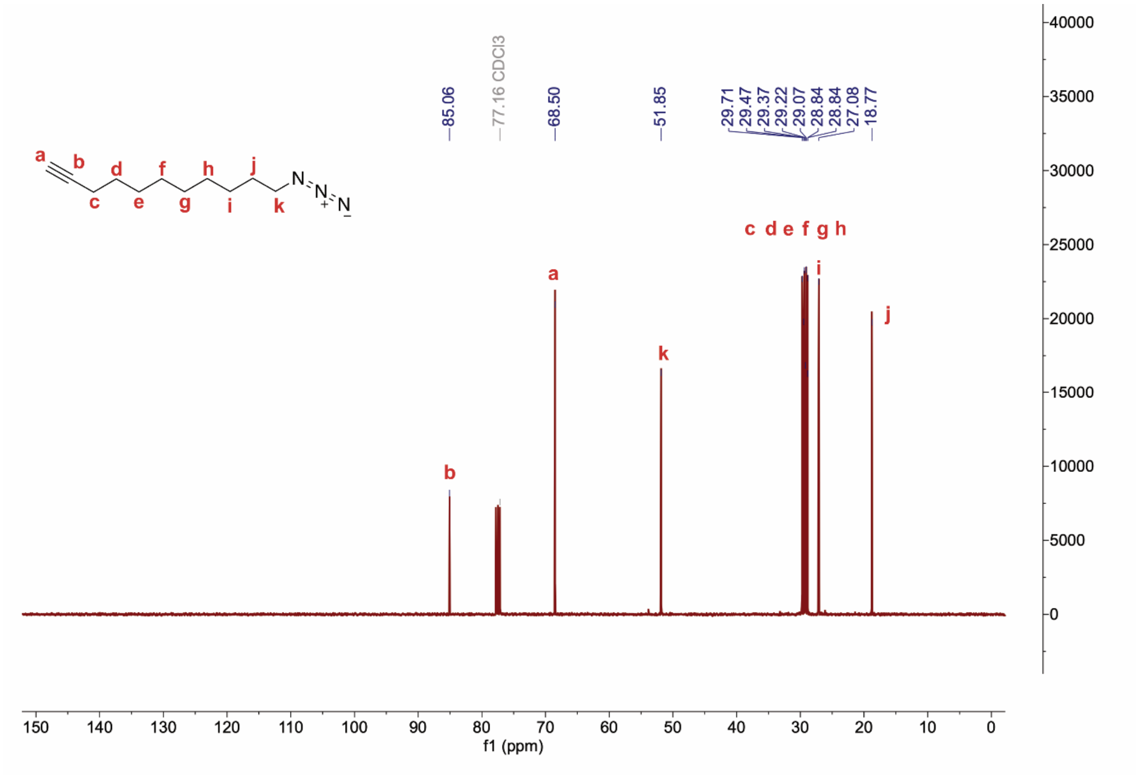

Figure S17: ^{13}C NMR spectrum of 11-azidoundec-1-yne ($CDCl_3$).

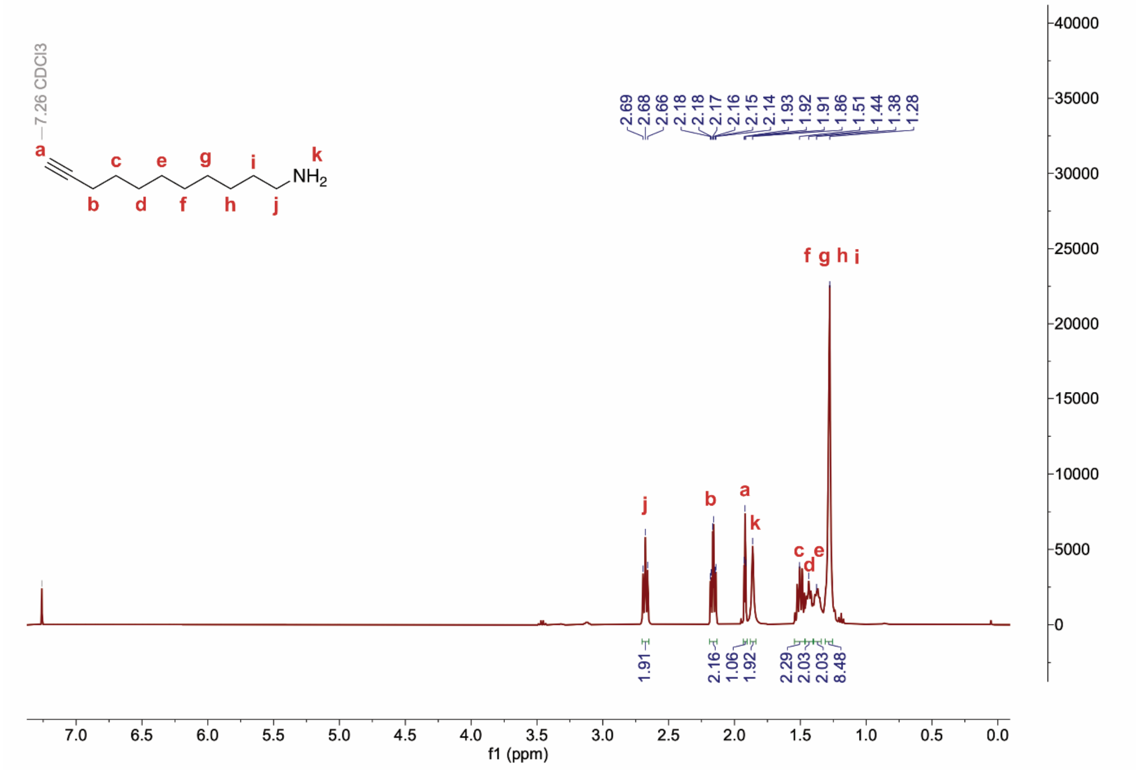

Figure S18: ^{1}H NMR spectrum of undec-10-yn-1-amine ($CDCl_3$).

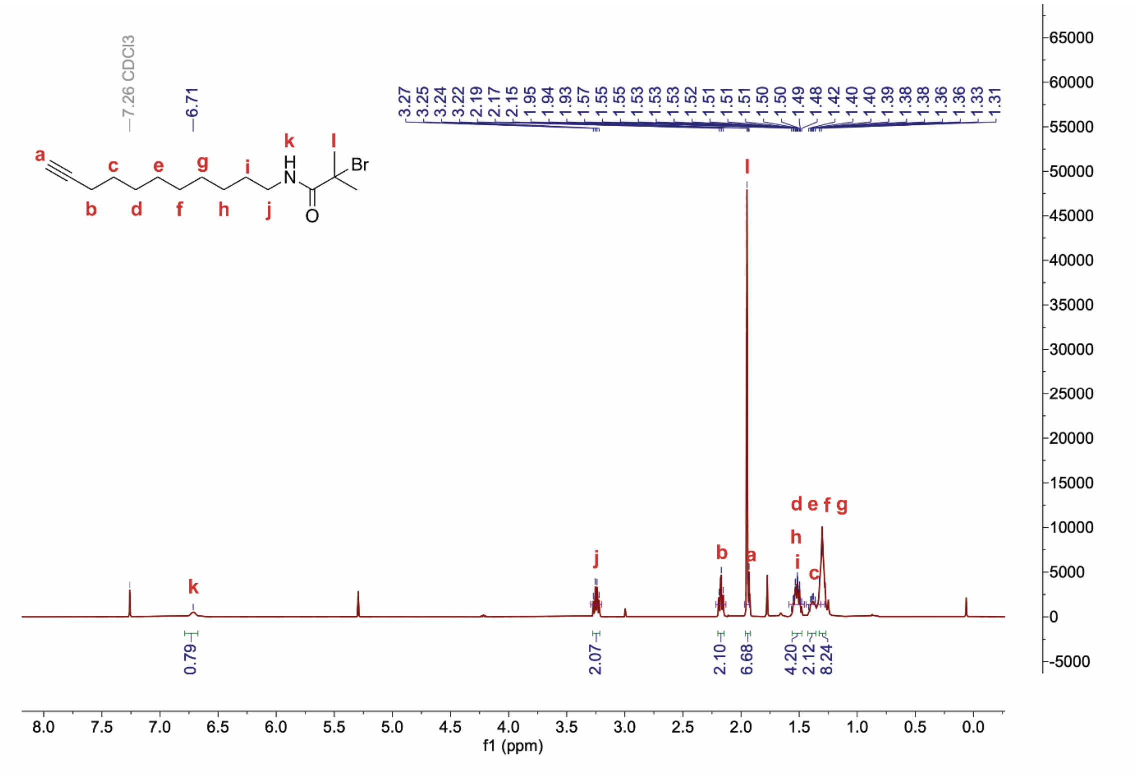

Figure S19: ^{1}H NMR spectrum of 2-bromo-2-methyl-N-(undec-10-yn-1-yl)propenamide ($CDCl_3$).

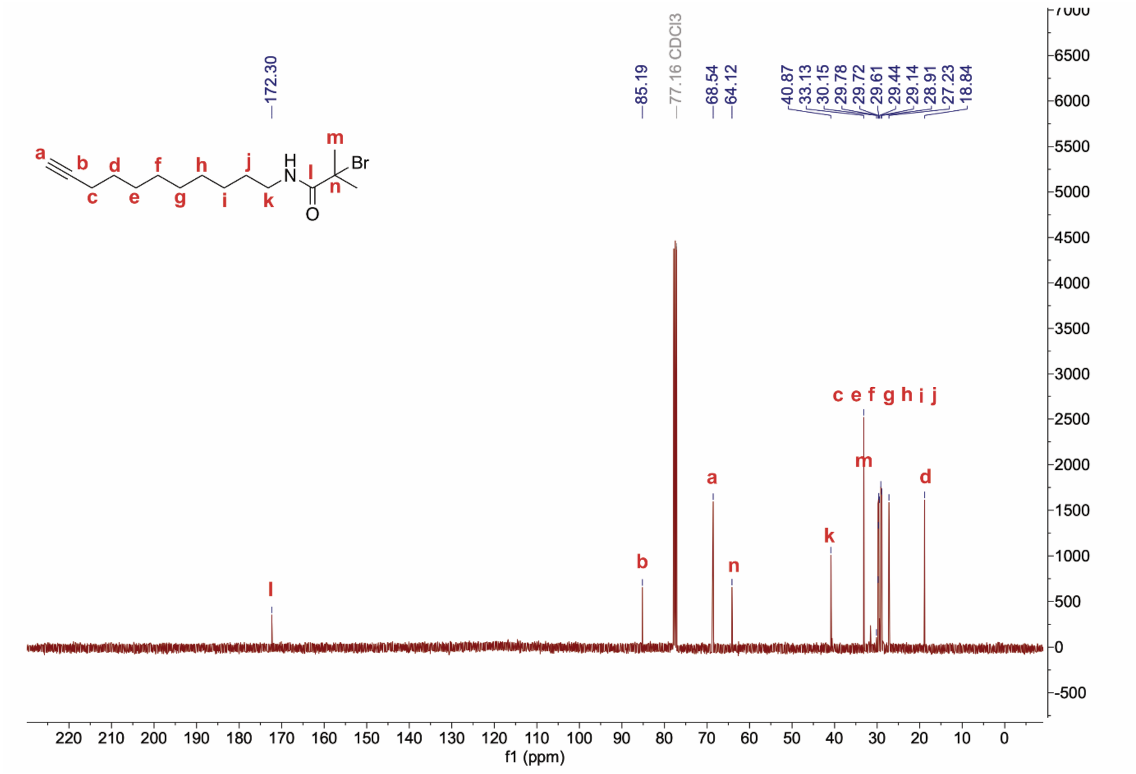

Figure S20: ^{13}C NMR spectrum of 2-bromo-2-methyl-N-(undec-10-yn-1-yl) propenamide ($CDCl_3$).

REFERENCES

[1] J. Wang, H. Klok, "Swelling-Induced Chain Stretching Enhances Hydrolytic Degrafting of Hydrophobic Polymer Brushes in Organic Media" *Angewandte Chemie* **2019**, *131*, 10094–10098.

[2] J. K. Chen, P. C. Pai, J. Y. Chang, S. K. Fan, "pH-responsive one-dimensional periodic relief grating of polymer brush-gold nanoassemblies on silicon surface" *ACS App. Mater. Interfaces* **2012**, *4*, 1935–1947.

[3] J. C. Grim, T. E. Brown, B. A. Aguado, D. A. Chapnick, A. L. Viert, X. Liu, K. S. Anseth, "A Reversible and Repeatable Thiol-Ene Bioconjugation for Dynamic Patterning of Signaling Proteins in Hydrogels" *ACS Cent. Sci.* **2018**, *4*, 909–916.

[4] G. Panzarasa, G. Soliveri, K. Sparnacci, S. Ardizzone, "Patterning of polymer brushes made easy using titanium dioxide: Direct and remote photocatalytic lithography" *Chem. Comm.* **2015**, *51*, 7313–7316.

[5] N. C. Ataman, H. A. Klok, "Degrafting of Poly(poly(ethylene glycol) methacrylate) Brushes from Planar and Spherical Silicon Substrates" *Macromolecules* **2016**, *49*, 9035–9047.

[6] A.-C. Bédard, S. K. Collins, "Advanced strategies for efficient macrocyclic Cu(I)-catalyzed cycloaddition of azides" *Org. Lett.* **2014**, *16*, 5286–5289.

[7] P. Waelès, C. Clavel, K. Fournel-Marotte, F. Coutrot, "Synthesis of triazolium-based mono- and tris-branched [1]rotaxanes using a molecular transporter of dibenzo-24-crown-8" *Chem. Sci.* **2015**, *6*, 4828–4836.

[8] S. Sant, H. A. Klok, "Linear, Y- and Ψ-shaped poly(2-(dimethylamino)ethyl methacrylate) and poly(methyl methacrylate) brushes prepared by surface-initiated polymerization from a homologous series of ATRP initiators" *Eur. Polym. J.* **2024**, *205*, DOI 10.1016/j.eurpolymj.2023.112706.

[9] F. K. Metze, I. Filipucci, H. A. Klok, "Supramolecular Polymer Brushes Grown by Surface-Initiated Atom Transfer Radical Polymerization from Cucurbit[7]uril-based Non-Covalent Initiators" *Angew. Chem. Int. Ed.* **2023**, *62*, DOI 10.1002/anie.202305930.

[10] T. Li, H. Li, H. Wang, W. Lu, M. Osa, Y. Wang, J. Mays, K. Hong, "Chain flexibility and glass transition temperatures of poly(n-alkyl (meth)acrylate)s: Implications of tacticity and chain dynamics" *Polymer (Guildf).* **2021**, *213*, DOI 10.1016/j.polymer.2020.123207.

[11] D. G. Mintis, M. Dompé, M. Kamperman, V. G. Mavrantzas, "Effect of Polymer Concentration on the Structure and Dynamics of Short Poly(N, N-dimethylaminoethyl methacrylate) in Aqueous Solution: A Combined Experimental and Molecular Dynamics Study" *Journal of Physical Chemistry B* **2020**, *124*, 240–252.
